# ICAM-1 identifies preadipocytes and restricts white adipogenesis by adhering immune cells

**DOI:** 10.1038/s41418-025-01551-2

**Published:** 2025-08-15

**Authors:** Chunxing Zheng, Jiayin Ye, Qian Yang, Keli Liu, Cheng Chen, Jianchang Cao, Qing Li, Yueqing Xue, Hui Ma, Arnold B. Rabson, Changshun Shao, Fei Hua, Lydia Sorokin, Gerry Melino, Yufang Shi, Ying Wang

**Affiliations:** 1 The Fourth Affiliated Hospital of Soochow University and Institutes for Translational Medicine, Soochow University, Suzhou, China; 2 Shanghai Institute of Nutrition and Health, University of Chinese Academy of Sciences, Chinese Academy of Sciences, Shanghai, China; 3 The Third Affiliated Hospital of Soochow University, Changzhou, and Institutes for Translational Medicine, Soochow University, Suzhou, China; 4 Department of Plastic Surgery, Zhongshan Hospital, Fudan University, Shanghai, China; 5 Jiangsu Yize biological Technology Co., LTD, Changzhou, China; 6 The Child Health Institute of New Jersey, Rutgers-Robert Wood Johnson Medical School, New Brunswick, NJ USA; 7 Institute of Physiological Chemistry and Pathobiochemistry and the Cells-in-Motion Interfaculty Centre (CIMIC), University of Muenster, Muenster, Germany; 8 Department of Experimental Medicine, TOR, University of Rome Tor Vergata, 00133 Rome, Italy

**Keywords:** Chronic inflammation, Metabolic disorders

## Abstract

Adipose stem cell hierarchy was delineated by scRNA-seq analysis, revealing that ICAM-1, a glycoprotein that mediates cell-cell interaction, is a preadipocyte marker. However, the cellular and molecular mechanisms of how ICAM-1^+^ preadipocytes contribute to adipose tissue homeostasis in vivo remain unclear. To address this, *Icam1*^*+/CreERT2*^ mice were generated, and it was demonstrated that ICAM-1-expressing progenitors actively participated in developing and remodeling white adipose tissue. Under a high-fat diet, both proliferation and adipogenic differentiation of ICAM-1^+^ preadipocytes increased significantly. Interestingly, ICAM-1 plays a critical role in maintaining the interaction between preadipocytes and immune cells, acting as a checkpoint on white adipogenesis. Mice lacking ICAM-1 specifically in stromal cells exhibited worsened hyperplastic obesity, showing heightened fatty acid synthesis and lipid storage in adipose tissue, and the related insulin resistance. In human adipose tissue, ICAM-1 also marked committed preadipocytes and mediated adhesion between preadipocytes and immune cells. Thus, our study shows that ICAM-1 marks preadipocytes and curbs adipogenesis by facilitating adhesion between preadipocytes and immune cells.

## Introduction

Adipose tissue is essential in maintaining energy balance [1, 2]. While research on adipose tissue has mostly focused on identifying the origin and differentiation mechanisms of adipocytes, there have been longstanding theories about the presence of progenitor cells in adipose tissue, dating back to the first micrograph of adipose tissue [3]. It is now understood that mesenchymal stem cells (MSCs) are the adipocyte precursor cells responsible for the sequential development of adipocyte progenitors and ultimately mature adipocytes. It is worth noting that MSCs also serve as progenitors of osteoblasts, meaning that their development involves both a lineage determination stage and a terminal differentiation stage [4].

Significant progress has been made in comprehending the homeostasis of adipose tissues, owing to the rapid advancement of stem cell biology and the development of lineage tracing technologies. Indeed, progress has been achieved in identifying and labeling adipose progenitors and tracing their de novo adipogenic processes. The stromal vascular fraction (SVF) derived from adipose tissue comprises a heterogeneous mixture of cell populations, such as MSCs, preadipocytes, endothelial cells, pericytes, T cells, and macrophages. CD31^−^CD45^−^Ter119^−^ SVF cells that express Sca-1, CD34, CD29, and CD24 are enriched in adipocyte progenitors and have been successfully used to reconstitute fat pads in lipodystrophic mice [5]. Further analysis showed that perivascular PDGFR-β^+^ cells in adipose tissue exhibit a high degree of adipogenic potential [6], while PDGFR-α^+^ mesenchymal progenitors are responsible for ectopic fat cell formation [7]. Our initial research revealed spontaneous adipogenic differentiation of CD31^−^CD45^−^Sca-1^+^ICAM-1^+^ adipose SVF cells, suggesting that committed preadipocytes reside within this population [8].

Single-cell RNA sequencing (scRNA-seq) now enables in vivo mapping of adipose progenitor cellular hierarchies and high-resolution identification of ICAM-1^+^ preadipocytes [9–12].

Most of our current understanding of the regulatory mechanisms of adipogenesis comes from in vitro studies using preadipocyte cell lines. These studies have shown that the transition from adipose progenitors to adipocytes is tightly regulated by multiple factors, such as soluble factors, membrane proteins, extracellular matrix, intracellular signal transducers, metabolites, and various transcription factors [13–16]. However, the tempo-spatial changes of preadipocytes during adipose tissue remodeling in vivo and the molecular mechanisms involved, including the potential role of ICAM-1 expressed on preadipocytes, are yet to be fully comprehended.

The immune milieu plays a crucial role in regulating tissue development, regeneration, and repair. Its direct impact on the fate and behavior of tissue progenitor cells, such as hair follicle stem cells, muscle satellite cells, mammary stem cells, and liver progenitors, has garnered much attention [17–20]. Adipose tissue is rich in immune cells that are believed to affect the mass and properties of obese adipose tissue. In a lean state, a significant number of immune cells, including macrophages and eosinophils, are present in the fat depots [21]. During obesity development, an influx of immune cells into adipose tissue fuels local inflammation [22]. Earlier in vitro studies have shed light on the impact of inflammatory factors on adipogenesis [23, 24]. However, unlike in the cell culture media, the diffusion of cytokines from immune cells to target cells in intact adipose tissues can be restricted by multiple barriers, including the limited extracellular space and binding to the extracellular matrix (ECM) [25, 26]. Therefore, it is imperative to explore the dynamics and molecular mechanisms of the preadipocyte-immune cell interactions during adipose tissue remodeling.

In this study, we used multiple lineage-tracing approaches to track the development of different cell lineages and found that adipocytes are differentiated from ICAM-1^+^ preadipocytes. This molecule helps preadipocytes adhere to immune cells and prevents their differentiation into adipocytes. When we disrupted this interaction between preadipocytes and immune cells, preadipocytes were able to differentiate into adipocytes, leading to hyperplastic obesity and its related insulin resistance. Therefore, ICAM-1 not only marks preadipocytes but also creates an immune environment for them to actively regulate adipose tissue homeostasis and remodeling.

## Results

### Genetic identification of ICAM-1^+^ preadipocytes in vivo

**Fig. 1:**
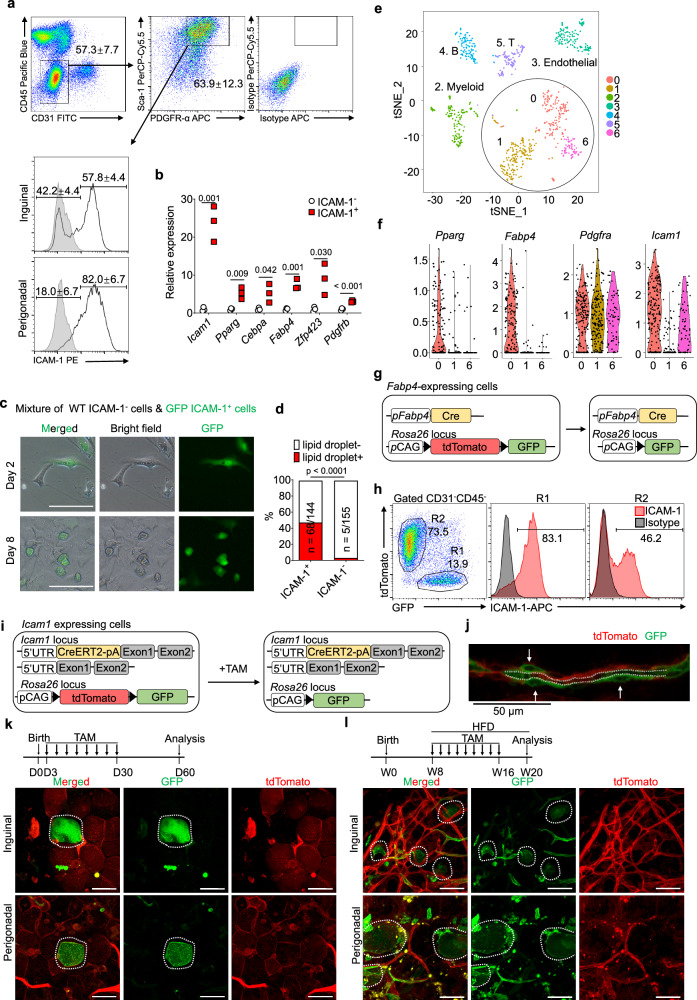
Adipogenesis of ICAM-1^+^ preadipocytes in vivo. **a** Representative flow cytometric plots showing that CD31^−^CD45^−^Sca-1^+^PDGFR-α^+^ adipose SVF cells were separated into ICAM-1^−^ and ICAM-1^+^ subsets. The numbers in plots indicate means ± s.d. of the percentages of gated cells, summarizing the results of four independent experiments conducted on nine mice. **b** Expression of *Icam1*, *Pparg*, *Cebpa*, *Fabp4, Zfp423* and *Pdgfrb* in CD31^−^CD45^−^Sca-1^+^PDGFR-α^+^ICAM-1^−^ and CD31^−^CD45^−^Sca-1^+^PDGFR-α^+^ICAM-1^+^ SVF cells sorted from inguinal adipose tissue of wild-type mice (n = 3 in each group, representative of two independent experiments). Unpaired, two-tailed *t*-test was performed. **c,d** ICAM-1^+^ cells spontaneously differentiate into adipocytes. CD31^−^CD45^−^ICAM-1^+^ SVF cells from GFP-transgenic mice and CD31^−^CD45^−^ICAM-1^−^ SVF cells from wild-type mice were co-cultured in regular medium. The formation of cytosolic lipid droplets was observed, and micrographs were taken on Days 2 and 8 (**c**). Scale bars, 100 μM. Percentages of ICAM-1^+^ and ICAM-1^−^ cells with or without lipid droplets on Day 8 (**d**). Two-tailed Fisher’s exact test was applied, representative of 2 independent experiments. **e** t-distributed stochastic neighbor embedding (tSNE) analysis of an adipose scRNA-seq dataset (GSE109774) identified three distinct subsets of ASCs labeled as cluster 0, 1, and 6. **f** The expression levels of *Pparg*, *Fabp4*, *Pdgfra*, and *Icam1* in each cluster of ASCs from the scRNA-seq dataset were shown in violin plots. **g** Diagrammatic representation of using *Fabp4*-Cre;mTmG mice for investigating lineage progression of preadipocytes during adipocyte development. **h** CD31^−^CD45^−^ SVF cells in the inguinal adipose tissue of *Fabp4*-Cre;mTmG mice were analyzed using flow cytometry, revealing that the majority of *Fabp4*-expressing cells were ICAM-1^+^, representative of 5 independent experiments. **i**. Diagrammatic representation of the generation of *Icam1*-tracing mice by crossing mTmG mice with *Icam1*^+/CreERT2^ mice. Recombinase activation was induced by administration of tamoxifen (TAM). **j** Whole-mount fluorescence imaging of the adipose tissue of tamoxifen-treated *Icam1*^*+/CreERT2*^;mTmG mice showing the ICAM-1-expressing cells in vasculature. The capillary lumen is outlined with dashed lines, and arrows indicate the perivascular ICAM-1^+^ cells. **k–l** Whole-mount fluorescence imaging of inguinal and perigonadal adipose tissue from *Icam1*^*+/CreERT2*^;mTmG mice showing the emergence of GFP^+^ adipocytes derived from ICAM-1-expressing preadipocytes upon tamoxifen induction either during postnatal development (**k**) or obesity development (**l**). Neonatal mice (**k**) and HFD-treated mice (**l**) were administered with tamoxifen at 30 μg/g body weight at the indicated time points. The dotted line circles indicate GFP^+^ mature adipocytes. Scale bars, 50 μM. Confocal Z-stack fluorescent images are presented, and 3-dimensional projections of these images are also shown in Videos S1–S4.

Understanding the hierarchy of adipocyte lineages is crucial for deciphering how adipose tissue homeostasis and remodeling are regulated. Using flow cytometric analysis, we found that the majority of the non-endothelial, non-hematopoietic (CD31^−^CD45^−^) SVF cells in perigonadal and inguinal adipose tissue expressed PDGFR-α and Sca-1 — established pan-markers for progenitors of mesenchymal lineages [27]. These cells could be further subdivided into two subsets, ICAM-1^+^ and ICAM-1^−^ cells (Fig. 1a). The canonical adipogenic genes, including *Pparg*, *Cebpa*, *Fabp4*, and *Zfp423*, were expressed at much higher levels in the ICAM-1^+^ subset than in the ICAM-1^−^ subset (Fig. 1b). To assess the adipogenic potential of these two subsets, CD31^−^CD45^−^ICAM-1^+^ SVF cells from GFP transgenic mice were cultured with CD31^−^CD45^−^ICAM-1^−^ SVF cells from wild-type (WT) mice at a 1:1 ratio without adipogenesis induction. Both cell types exhibited a similar spindle-shaped morphology on day 2. After eight days, a significant accumulation of lipid droplets can be observed in CD31^−^CD45^−^GFP^+^ICAM-1^+^ cells, but not in CD31^−^CD45^−^ICAM-1^−^ cells, indicating that CD31^−^CD45^−^ICAM-1^+^ SVF cells could spontaneously differentiate into adipocytes (Fig. 1c, d). This result was also observed in a reciprocal co-culture of CD31^−^CD45^−^ICAM-1^+^ SVF cells from WT mice and CD31^−^CD45^−^ICAM-1^−^ SVF cells from GFP mice (Fig. S1a, b). Therefore, CD31^−^CD45^−^ICAM-1^+^ SVF cells exhibit an enhanced propensity for adipogenic differentiation.

We also analyzed single-cell RNA sequencing (scRNA-seq) data from a published dataset (GSE109774) derived from adipose SVF of subcutaneous fat tissues [12] and identified seven clusters using t-SNE. The SVF clusters were identified as hematopoietic lineage cells, endothelial cells, and adipose stromal cells (ASCs) (Fig. 1e, Fig. S1c–f, Table S1). ASCs are non-hematopoietic, non-endothelial SVF cells that encompass preadipocytes. We compared the marker genes of the three ASC clusters with those upregulated during early stages of adipogenesis in in vitro cultured preadipocytes (GSE29899) [28] and found that cluster 0 possessed more adipogenic genes than clusters 1 and 6 (Fig. S1g, h). Gene ontology analysis revealed that the highlighted adipogenic genes in cluster 0 mainly belonged to the lipid metabolism pathways (Fig. S1i–l). Cells in cluster 0 were found to exclusively express *Pparg* and *Fabp4*, two well-known adipogenic genes, indicating that they are committed preadipocytes (Fig. 1f). Through analyzing the marker genes that encode cell surface proteins, we discovered that *Icam1* was the most abundantly and preferentially expressed gene in cells of cluster 0 (Table S2). Unlike *Pdgfra*, a pan-marker expressed evenly on all three ASC clusters, *Icam1* was highly expressed on cells of cluster 0, negatively expressed on cells of cluster 1, and moderately expressed on cells of cluster 6 (Fig. 1f). To further refine the identification of cluster 0, we examined the expression levels of surface markers and found that CD36 or Notch1 can be combined with ICAM-1 to pinpoint cluster 0 in CD31^−^CD45^−^ SVF cells (Fig. S1m–q). Together, in line with a scRNA-seq-based study that revealed the hierarchy of adipose precursors [9], we demonstrated that committed preadipocytes are ICAM-1^+^ ASCs.

We next employed lineage tracing strategies to investigate the expression of ICAM-1 on preadipocytes. As *Fabp4* is selectively expressed on cells of the adipocyte lineage, we crossed *Fabp4*-Cre transgenic mice with mTmG reporter mice [29, 30] (Fig. 1g, S2a). In *Fabp4*-Cre;mTmG mice, cells expressing GFP indicate current or past expression of *Fabp4*. We isolated the SVF cells from adipose tissues of these mice and analyzed them by flow cytometry. The CD31^−^CD45^−^ SVF cells were divided into GFP^+^ (R1) and tdTomato^+^ (R2) populations (Fig. 1h, Fig. S2b, c). The GFP^+^ cells are the Fabp4-expressing cells and match cluster 0 of the cells analyzed by scRNA-seq. Importantly, this population highly expressed ICAM-1 (Fig. 1h). The tdTomato^+^ cells were further divided into ICAM-1^−^ and ICAM-1^+^ subpopulations, which likely correspond to *Icam1* negatively expressing cluster 1 and *Icam1* moderately expressing cluster 6 of the scRNA-seq analyzed cells, respectively. Together with our scRNA-seq analysis, these lineage tracing results corroborated the notion that ICAM-1^+^ ASCs include committed preadipocytes. The GFP^+^ cells in perigonadal adipose tissue were also predominantly ICAM-1^+^ (Fig. S2c). Unlike inguinal adipose tissue, the tdTomato^+^ cells in perigonadal adipose tissue were mainly positive for ICAM-1, indicating a lack of ICAM-1^−^ ASCs in this adipose depot. In rodents, white adipose tissue develops predominantly postnatally, particularly during early postnatal life [2]. We found that these GFP^+^ cells were distinctly detected at postnatal day 18 (P18) and P30 in *Fabp4*-Cre;mTmG mice (Fig. S2d). Importantly, these GFP^+^ cells primarily express ICAM-1 (Fig. S2d), indicating that ICAM-1 serve as a valid marker for adipogenic progenitors in newborn mice.

To determine whether ICAM-1^+^ cells could give rise to mature adipocytes in vivo, we generated mice with CreERT2 knock-in at the *Icam1* locus (Fig. S2e, f). These mice were then crossed with the mTmG mice. Upon tamoxifen administration, cells that express or had expressed *Icam1* could be identified by the expression of GFP (Fig. 1i). Interestingly, most of these GFP^+^ cells are localized to the vasculature, including endothelial cells, which are well-known for the expression of ICAM-1, as well as perivascular cells (Fig. 1j). When tamoxifen was administrated to mice during their neonatal stage, GFP^+^ adipocytes were detected in both subcutaneous adipose tissue and visceral adipose tissue after they became adult (Fig. 1k, Video S1, S2). When tamoxifen was administered at the initiation of high-fat diet (HFD)-induced obesity in adult mice, GFP^+^ adipocytes were detected in obese adipose tissue (Fig. 1l, Video S3, S4). Notably, the expression of ICAM-1 on preadipocytes gradually vanished during adipogenesis and was undetectable in mature adipocytes (Fig. S2g, h). Therefore, ICAM-1 marks preadipocytes that give rise to adipocytes postnatally and contribute to the expansion of adipose tissue during obesity. Most of the newly generated GFP^+^ adipocytes were found to be proximate to vasculature, consistent with the perivascular localization of ICAM-1^+^ ASCs. Taken together, ICAM-1^+^ ASCs are bona fide preadipocytes that give rise to mature adipocytes in vivo.

### ICAM-1^+^ preadipocytes actively proliferate and differentiate during obesity induction

**Fig. 2:**
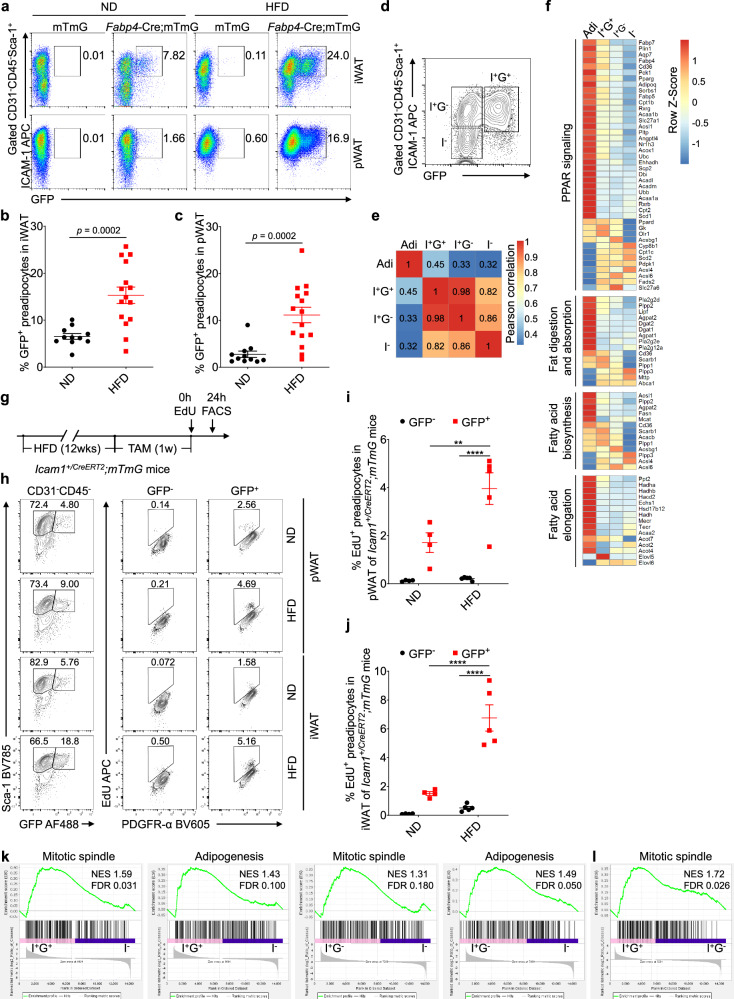
ICAM-1^+^ preadipocytes differentiate and proliferate in vivo upon an HFD. **a-c** Flow cytometric analysis (**a**) and statistical summaries of ICAM-1^+^GFP^+^ preadipocytes in inguinal (iWAT) (**b**) and perigonadal (pWAT) white adipose tissue (**c**) isolated from *Fabp4*-Cre;mTmG mice that were subjected to either a normal or high-fat diet. Data were pooled from the mice analyzed in three independent experiments. Significance was analyzed using unpaired *t*-test with Welch’s correction to account for unequal variances between groups. **d** The strategy of sorting ICAM-1^+^GFP^+^ (I^+^G^+^), ICAM-1^+^GFP^−^ (I^+^G^−^) and ICAM-1^−^ (I^−^) subsets of ASCs from *Fabp4*-Cre;mTmG mice that were treated with HFD, followed by RNA-sequencing analysis. **e** Heatmap of Pearson correlation of total gene expression in adipocytes (Adi), I^+^G^+^, I^+^G^−^, and I^−^ cells, as defined in **d.** The numbers in color blocks indicate the values of the respective Pearson’s *r*. **f** Heatmap showing differentially expressed adipocyte-related genes in the PPAR signaling, fat digestion and absorption, fatty acid biosynthesis, and fatty acid elongation pathways in Adi, I^+^G^+^, I^+^G^−^, and I^−^ cells. Cutoff was set as |log_2_ (foldchange)|>1 & FDR < 0.01. **g-j** *Icam1*^*+/CreERT2*^;mTmG mice were fed ND or HFD for 12 weeks, given a diet with 0.1% Tamoxifen for one week, and then administered 10 ug/g EdU (**g**). Flow cytometric analysis was conducted 24 h later. Representative flow cytometric plots (**h**) and statistical summaries of EdU incorporation in GFP^+^ and GFP^−^ populations of CD31^−^CD45^−^Sca-1^+^ SVF cells from perigonadal (pWAT) (**i**) and inguinal white adipose tissue (iWAT) (**j**) are presented. **k–l** Gene Set Enrichment Analysis (GSEA) was performed on RNA-seq data to compare the ortholog hallmark gene sets, including mitotic spindle and adipogenesis, among ICAM-1^+^GFP^+^ (I^+^G^+^), ICAM-1^+^GFP^−^ (I^+^G^−^), and ICAM-1^−^ (I^−^) subsets of ASCs from HFD-treated *Fabp4*-Cre;mTmG mice. NES, normalized enrichment score; FDR, false discovery rate.

Since ICAM-1^+^ preadipocytes are the major cell population to generate adipocytes in vivo, we further examined the dynamics of ICAM-1^+^ preadipocytes in adipose tissue during obesity induction. To achieve this, *Fabp4*-Cre;mTmG mice were fed with an HFD, and the GFP^+^ cell population was analyzed in the adipose tissue of both lean mice and obese mice. In lean mice, we found that a small fraction of CD31^−^CD45^−^Sca-1^+^ICAM-1^+^ cells were GFP positive, reflecting the basal level of adipocyte turnover (Fig. 2a). Strikingly, in HFD-treated mice, the proportion of GFP^+^ cells in CD31^−^CD45^−^Sca-1^+^ICAM-1^+^ cells were greatly increased in both inguinal and perigonadal adipose tissues (Fig. 2a–c), demonstrating that *Fabp4* transcription in ICAM-1^+^ preadipocytes was elevated during obesity development.

Next, we isolated mature adipocytes (Adi), CD31^−^CD45^−^Sca-1^+^ICAM-1^+^GFP^+^(I^+^G^+^), CD31^−^CD45^−^Sca-1^+^ICAM-1^+^GFP^−^ (I^+^G^−^), and CD31^−^CD45^−^Sca-1^+^ICAM-1^−^ (I^−^) SVF cells from the inguinal adipose tissue of obese mice (Fig. 2d) for bulk RNA-seq analysis. We found that the overall gene expression profile of the ICAM-1^+^GFP^+^ subset closely resembled that of the ICAM-1^+^GFP^−^ subset, with a high correlation coefficient of 0.98 (Fig. 2e). Compared to the other two subsets, ICAM-1^+^GFP^+^ cells showed a stronger correlation with adipocytes in their overall gene expression patterns (Fig. 2e). We specifically analyzed the differentially expressed genes among different cell populations involved in PPAR signaling, fat digestion and absorption, fatty acid biosynthesis, and fatty acid elongation, which are key biological processes in mature adipocytes. We found that most of these genes enriched in adipocytes can be observed in ICAM-1^+^GFP^+^ subset and, to a lesser extent, in ICAM-1^+^GFP^−^ subset, but not in ICAM-1^−^ cells (Fig. 2f), suggesting that ICAM-1^+^GFP^+^ ASCs are precursors committed to generating adipocytes during obesity. To further delineate the hierarchy of the two ICAM-1^+^ cell subsets, we sorted the CD31^−^CD45^−^ICAM-1^+^ SVF cells from *Fabp4*-Cre;mTmG mice, which consisted of both tdTomato^+^ and GFP^+^ cells. Upon exposed to the adipogenic induction medium, the GFP^+^ cells rapidly assumed a round cellular morphology and differentiated into mature adipocytes (Fig. S3a, b). Importantly, GFP^+^tdTomato^+^ intermediate cells also appeared during this process (Fig. S3a, b), supporting a shift of ICAM-1^+^tdTomato^+^ cells to ICAM-1^+^GFP^+^ cells during adipogenesis. Together, these results suggest that under obese conditions, ICAM-1^+^ ASCs actively develop into ICAM-1^+^ *Fabp4*-expressing cells and subsequently acquire adipocyte features. These ICAM-1^+^ *Fabp4*-expressing cells can be referred to as incipient adipocytes.

Upon exposure to HFD-induction, the total ICAM-1^+^ population in CD31^−^CD45^−^Sca-1^+^ SVF cells was dramatically increased (Fig. 2a). This enhancement could be attributed to either a transition from ICAM-1^−^ cells to ICAM-1^+^ cells or an increase in proliferation of ICAM-1^+^ cells. The derivation of ICAM-1^+^ preadipocytes from DDP4^+^ multiple potent progenitor cells has been indicated [9], however, whether ICAM-1^+^ preadipocytes have the capacity to proliferate and expand in vivo during obesity remains obscure. To address this question, we subjected *Icam1*^*+/CreERT2*^*;mTmG* mice to an HFD to induce obesity, and we traced the ICAM-1^+^ preadipocytes upon tamoxifen administration. We then used the EdU (5-ethynyl-2’-deoxyuridine) incorporation assay to detect the proliferation of ASCs in adipose tissue (Fig. 2g). After administering EdU for 24 h, the incorporation of EdU in CD31^−^CD45^−^Sca-1^+^GFP^−^ SVF cells was barely detectable. In contrast, approximately 2% of GFP^+^ counterparts had EdU incorporation in adipose tissue of normal diet (ND)-treated mice (Fig. 2h–j). Upon HFD treatment, the EdU incorporation in GFP^+^ ASCs was significantly higher than that in GFP^+^ cells from lean mice, while the proliferation of ICAM-1^−^ cells was similar to that of ICAM-1^−^ cells from lean mice (Fig. 2h–j). These results suggest that ICAM-1^+^ ASCs, rather than ICAM-1^−^ ones, are actively cycling, which is further promoted upon HFD exposure (Fig. 2h–j). Similarly, utilization of HFD treated *Fabp4*-Cre;mTmG mice, we found that pathway enrichment analysis of transcriptome of ICAM-1^+^GFP^−^ and ICAM-1^+^GFP^+^ ASCs showed a close correlation to mitosis and adipogenesis (Fig. 2k). Notably, the mitotic spindle pathway was more enriched in ICAM-1^+^GFP^+^ cells than that in ICAM-1^+^GFP^−^ cells, suggesting a greater potential of ICAM-1^+^GFP^+^ ASCs to proliferate under the HFD condition (Fig. 2l). Taken together, ICAM-1^+^ preadipocytes can self-renew and actively proliferate to generate adipocytes during obesity development.

### ICAM-1 mediates the adhesion between preadipocytes and immune cells

**Fig. 3:**
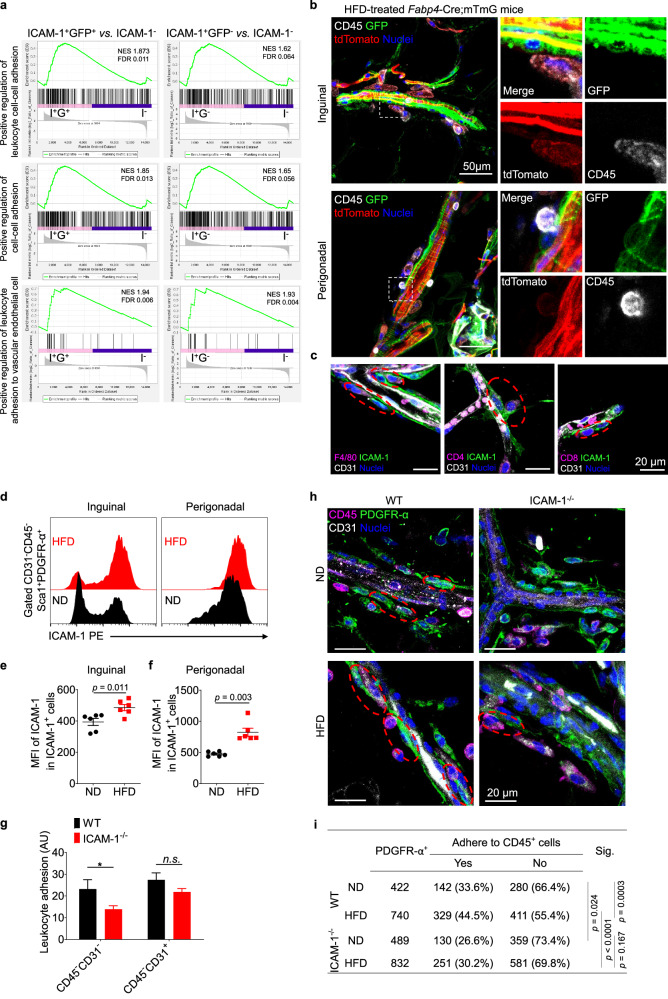
ICAM-1 is critical for the adhesion between preadipocytes and immune cells. **a** RNA-seq data from ICAM-1^+^GFP^+^ (I^+^G^+^), ICAM-1^+^GFP^−^ (I^+^G^−^), and ICAM-1^−^ (I^−^) subsets of ASCs from HFD-treated Fabp4-Cre;mTmG mice were used to perform GSEA on ontology gene sets, including positive regulation of leukocyte cell-cell adhesion (top), positive regulation of cell-cell adhesion (middle), and positive regulation of leukocyte adhesion to vascular endothelial cell (bottom). NES, normalized enrichment score; FDR, false discovery rate. **b** Representative fluorescent micrographs showing the adhesion between immune cells and perivascular GFP^+^ ASCs in inguinal and perigonadal adipose tissues of HFD-treated Fabp4-Cre;mTmG mice. The areas highlighted in dashed rectangles (left) were zoomed in and with separate fluorescent channels displayed (right). **c** Representative fluorescent micrographs showing the adhesion of macrophages (F4/80^+^, left), CD4^+^ T cells (CD4^+^, middle) and CD8^+^ T cells (CD8^+^, right) to ICAM-1^+^ preadipocytes in adipose tissue. Blood vessels were stained with CD31, and nuclei were counterstained with Hoechst33342. **d-f** Representative flow cytometric plots (**d**) and statistical analysis (**e**, **f**) of ICAM-1^+^ preadipocytes and the expression level of ICAM-1 on these cells from mice on normal or high-fat diet. MFI, mean fluorescence intensity. Unpaired, two-tailed *t*-test was performed. Results are representative of three independent experiments. **g** In vitro leukocyte adhesion assay of WT and ICAM-1^−/−^ ASCs showing the dependence of ICAM-1 for adhesion of immune cells to ASCs. The OD values (arbitrary units, AU) of CFSE-labeled leukocytes adhering to CD45^−^CD31^−^ ASCs or CD45^−^CD31^+^ endothelial cells were shown as means ± SEM (n = 8 wells in each group, representative of two independent experiments). *p < 0.05, two-way ANOVA followed by Sidak’s multiple comparison test. **h-i **Representative fluorescent micrographs (**h**) and statistical analysis (**i**) of the adhesion events between CD45^+^ immune cells and CD45^−^CD31^−^PDGFR-α^+^ ASCs in adipose tissue of ND or HFD-treated WT and ICAM-1^−/−^ mice. Cell enumeration was performed on confocal images of whole-mount staining of adipose tissue from 5 mice in each group (about 12 representative image files per mouse). Two-tailed Fisher’s exact test was performed.

To parse out key factors in controlling ICAM-1^+^ preadipocyte commitment to differentiation, we analyzed differences among ICAM-1^+^GFP^+^, ICAM-1^+^GFP^−^ and ICAM-1^−^ ASCs of HFD-treated *Fabp4*-reporter mice using RNA-seq. A significant enrichment of the activation of the pathways related to positive regulation of cell-cell adhesion, leukocyte cell-cell adhesion, and leukocyte adhesion to vascular endothelial cells was observed in both ICAM-1^+^GFP^+^ and ICAM-1^+^GFP^−^ ASCs, as compared to ICAM-1^−^ ASCs (Fig. 3a). Meanwhile, scRNA-seq analysis of the three populations in naïve mice revealed that the genes encoding cell adhesion molecules (CAMs) were also highly expressed in the committed preadipocyte cluster (cluster 0), but not in clusters 1 and 6 (Fig. S4a). Along with their location to blood vessels, the ICAM-1^+^ committed preadipocytes are highly possible to possess a strong potential to adhere to leukocytes.

During obesity development, the remodeling of adipose tissue is accompanied by elevated inflammation, characterized by extensive accumulation of innate and adaptive immune cells [31]. To verify the interaction between ICAM-1^+^ preadipocytes and immune cells, we examined the location of ICAM-1^+^ *Fabp4*-expressing incipient adipocytes in situ and their spatiotemporal position with immune cells in the obese adipose tissue of *Fabp4*-Cre;mTmG mice subjected to an HFD. Like ICAM-1^+^ preadipocytes (Fig. 1j), the majority of Fabp4-expressing cells were found to closely locate around the vasculature (Fig. 3b), indicating that the generation of mature adipocytes occurs in the perivascular niche. Surprisingly, we found that a number of the Fabp4-expressing incipient adipocytes (GFP^+^) interacted with CD45^+^ immune cells in obese adipose tissue of HFD-fed mice (Fig. 3b). To further investigate the details of this observation, we performed immuno-staining of committed preadipocytes with ICAM-1, endothelial cells with CD31, and various immune cells with their respective markers. We found that perivascular ICAM-1^+^ preadipocytes tightly co-localized with macrophages (F4/80^+^), CD4^+^ T cells (CD4^+^), and CD8^+^ T cells (CD8^+^) (Fig. 3c). These results reveal a likely physical association between ICAM-1^+^ committed preadipocytes and immune cells. In addition, these CD31^−^CD45^−^ ASCs are the primary source of chemokines, including CCL2 [21], CCL7, CXCL1, and CCL11, which are responsible for recruiting immune cells to obese adipose tissue (Fig. S4b–e).

ICAM-1 and VCAM-1 are the primary CAMs responsible for leukocyte adhesion under various pathophysiological conditions. The expression of ICAM-1 in committed preadipocytes was significantly higher than that of VCAM-1 (Fig. S4f), and the ICAM-1 level was further increased in obese adipose tissue (Fig. 3d–f). To determine the role of ICAM-1 in the heterotypic preadipocyte-immune cell adhesion, we performed a modified leukocyte adhesion assay, in which CD45^−^CD31^−^ ASCs from HFD-treated WT mice or ICAM-1^−/−^ mice were isolated and leukocyte adhesion to these stromal cells was measured. As a control, we also performed this assay with CD45^−^CD31^+^ endothelial cells from the adipose tissue of the same mice. Our results revealed that without ICAM-1, adhesion between ASCs and leukocytes is reduced. However, no notable differences were observed in leukocyte adhesion to endothelial cells with or without ICAM-1 (Fig. 3g), highlighting a unique role of ICAM- in facilitating immune cell adhesion to preadipocytes.

We next examined whether ICAM-1 is necessary for preadipocyte-immune cell adhesion in vivo. By immuno-staining of PDGFR-α, a pan-marker of preadipocytes, as well as CD45 and CD31 in adipose tissue, we found extensive attachments of CD45^+^ immune cells to PDGFR-α^+^ cells in WT mice. Such attachment was enhanced in the adipose tissue of HFD-treated mice. Conversely, ICAM-1 deficiency reduced the adhesion between PDGFR-α^+^ cells and CD45^+^ cells in both lean and obese adipose tissue (Fig. 3h). We counted a total of 2,483 PDGFR-α^+^ preadipocytes from both WT and ICAM-1^−/−^ mice and demonstrated that ICAM-1 is important in mediating the attachment of preadipocytes to immune cells (Fig. 3i). Notably, we found no significant difference in the infiltration of total CD45^+^ immune cells and their different subsets in adipose tissue of WT mice and ICAM-1^−/−^ mice fed with an HFD (Fig. S4g-j). Therefore, ICAM-1 is a key molecule mediating the adhesion between preadipocytes and immune cells in adipose tissue.

### ICAM-1 is a checkpoint in restricting preadipocyte fate

**Fig. 4:**
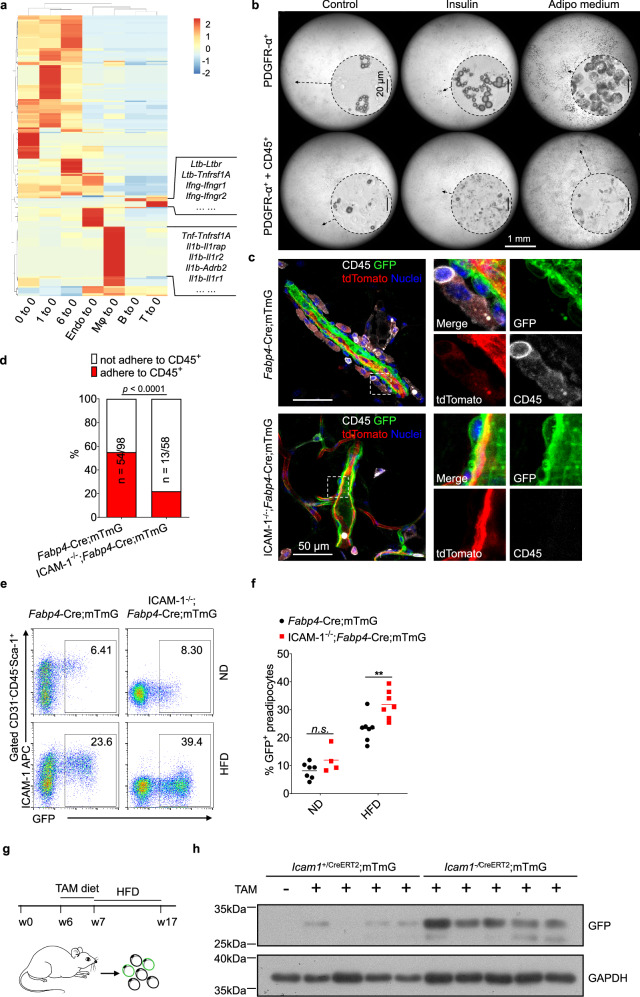
Immune cells attached to preadipocytes are anti-adipogenic. **a** Heatmap showing the activity potential of various cell populations (cluster 0–6) on committed preadipocytes (cluster 0), based on the expression levels of ligands from other clusters and their corresponding receptors on cluster 0. The representative ligands carried by macrophages, B cells, and T cells, along with the respective receptors presenting on cluster 0, were indicated. **b** The spontaneous and induced adipogenesis of sorted PDGFR-α^+^ preadipocytes when cultured with or without sorted CD45^+^ immune cells from adipose tissue of HFD-treated mice. The lipid droplets were manifested by Oil-red O staining. Scar bars, 20 μm for high magnification and 500 μm for low magnification. Representative result of two independent experiments is shown.** c-d** Representative fluorescent micrograph (**c**) and statistical analysis (**d**) of the adhesion between CD45^+^ immune cells and GFP^+^ perivascular cells in adipose tissue of *Fabp4*-Cre;mTmG and ICAM-1^−/−^;*Fabp4*-Cre;mTmG mice subjected to HFD (two-tailed Fisher’s exact test). Cell enumeration was performed on confocal images of whole-mount staining of adipose tissue from 2 mice in each group (about 12 representative image files per mouse). **e–f** Representative plots and statistical analysis of flow cytometry showing the increase of GFP^+^ incipient adipocytes in the HFD-treated *Fabp4*-Cre;mTmG mice in the absence of ICAM-1 (representative of three independent experiments). **p < 0.01, two-way ANOVA followed by Sidak’s multiple comparison test. **g-h** *Icam1*^+/CreERT2^;mTmG and *Icam1*^−^^/CreERT2^;mTmG mice were pulsed with tamoxifen-containing diet prior to induction of obesity with HFD (**g**), and their adipocytes were then isolated for immunoblotting of GFP (**h**) which served as a marker for the newly emerged GFP^+^ adipocytes (representative of two independent experiments).

Our histological examinations revealed that ICAM-1^+^ preadipocytes reside in a stromal cell niche composed of endothelial cells, different populations of ASCs, and various types of immune cells. To determine the signals potentially transmitted from neighboring cells to regulate committed preadipocytes, we profiled the ligand-receptor interactome based on the scRNA-seq data using an established strategy [30, 31]. We found that the ligands from different ASC clusters (1, 6, and 0 per se) to committed preadipocytes (cluster 0) exhibited similar patterns, characterized by multiple ECM proteins (Fig. 4a and Table S3). Endothelial cells mainly provided PDGFs to the PDGFR-α^+^PDGFR-β^+^ preadipocytes, which likely account for the preadipocytes’ perivascular location (Fig. 4a and Table S3). We also observed that immune cells predominantly interacted with preadipocytes via inflammatory cytokines, such as lymphotoxin-β produced by B cells, IFN-γ by T cells, and TNF-α and IL1-β by macrophages. The preadipocytes expressed abundant cognate receptors for these cytokines (Fig. 4a and Table S3), and in vitro studies have shown that they have anti-adipogenic effects [23, 24, 32]. This suggests that the immune cells may function to suppress preadipocyte differentiation.

To examine the impact of these immune cells on preadipocytes, we isolated CD31^−^CD45^−^PDGFR-α^+^ pan-preadipocytes from adipose tissue and subjected them to an adipogenic differentiation assay in the presence or absence of leukocytes from adipose tissue of mice fed an HFD. We observed that leukocytes markedly suppressed adipogenesis of PDGFR-α^+^ cells induced either by an adipogenic medium (a cocktail containing insulin, indomethacin, dexamethasone, and IBMX) or insulin alone (Fig. 4b). Additionally, the presence of leukocytes completely prevented spontaneous adipogenesis of these progenitors cultured in regular medium (Fig. 4b).

We next asked whether ICAM-1 on preadipocytes plays a role in mediating their adhesion to leukocytes and acts as a checkpoint to regulate preadipocyte differentiation. To test this hypothesis, we crossed the *Fabp4* reporter mice with ICAM-1^−/−^ mice and fed the resulting ICAM-1^−/−^;*Fabp4*-Cre;mTmG mice and their *Fabp4*-Cre;mTmG littermates an HFD. Consistent with our previous results (Fig. 3h, i), the interactions between CD45^+^ immune cells and GFP^+^ *Fabp4*-expressing cells were reduced in ICAM-1^−/−^;*Fabp4*-Cre;mTmG mice (Fig. 4c, d). Interestingly, ICAM-1 deficiency significantly increased the proportion of CD31^−^CD45^−^Sca-1^+^FABP4^+^ SVF cells in adipose tissue (Fig. 4e, f), suggesting that without ICAM-1 and its adhesion to immune cells, more committed preadipocytes differentiate to Fabp4-expressing early-stage adipocytes.

ICAM-1 cognately ligates to β2 integrin composed counterreceptors, including Mac-1 (integrin α_M_β_2_, CD11b/CD18) and LFA-1 (integrin α_L_β_2_, CD11a/CD18), to exert the cell adhesion effects [32]. In adipose tissue, integrin α_M_β_2_ is selectively expressed by myeloid cells, while integrin α_L_β_2_ is primarily expressed by T cells (Fig. S5a–c). Macrophages are the most abundant immune cells in adipose tissue (Fig. S4h, S4j) and highly express integrin α_M_ (Fig. S5d). We found that in integrin α_M_-deficient mice, there was no reduction in the cellularity of either total CD45^+^ immune cells or F4/80^+^ macrophages in adipose tissue (Fig. S5e), whereas these integrin α_M_-deficient macrophages showed decreased capacity to adhere to ASCs (Fig. S5f). Interestingly, integrin α_M_-deficient mice exhibited increased body weight and adiposity when subjected to an HFD (Fig. S5g–i). Intriguingly, such obesity was associated with reduced adipocyte size (Fig. S5j), indicating more adipocytes were generated in the absence of integrin α_M_. These data corroborate that the close interaction between preadipocytes and immune cells, mediated by the ligation of ICAM-1 to integrin α_M_β_2_, inhibits the adipogenesis of preadipocytes.

To further elucidate the role of ICAM-1 in regulating the generation of mature adipocytes, we crossed ICAM-1^−/−^ mice with *Icam1*^*+/CreERT2*^;mTmG mice to acquire *Icam1*^−^^*/CreERT2*^;mTmG mice and their *Icam1*^*+/CreERT2*^;mTmG littermates. Since knock-in of CreERT2 at the *Icam1 locus* results in null expression of ICAM-1, the *Icam1*^−^^*/CreERT2*^ mice do not express ICAM-1 and are equivalent to the ICAM-1^−/−^ mice. These *Icam1*^−^^*/CreERT2*^;mTmG mice and their *Icam1*^*+/CreERT2*^;mTmG littermates were fed a tamoxifen-containing diet for 7 days, followed by an HFD for 10 weeks (Fig. 4g). We then analyzed GFP expression in isolated mature adipocytes as an indicator of newly generated adipocytes. Our results showed that GFP expression was markedly higher in adipose tissue from *Icam1*^−^^*/CreERT2*^;mTmG mice compared to their *Icam1*^*+/CreERT2*^;mTmG littermates (Fig. 4h and Scan S1). Since the mTmG cassette is in the *Rosa26* locus [30], ensuring ubiquitous and uniform reporter protein expression, the increased GFP expression indicates increased newly generated adipocytes. These findings suggest that the absence of ICAM-1 enhances the adipogenic differentiation of preadipocytes. In this context, ICAM-1 functions as a checkpoint, maintaining the preadipocyte state and restricting adipogenic progression, especially under overnutrition conditions. In summary, our findings support a model in which the interaction between ICAM-1 and immune cells negatively regulates preadipocyte differentiation.

### ICAM-1 deficiency in stromal cells results in hyperplastic obesity

**Fig. 5:**
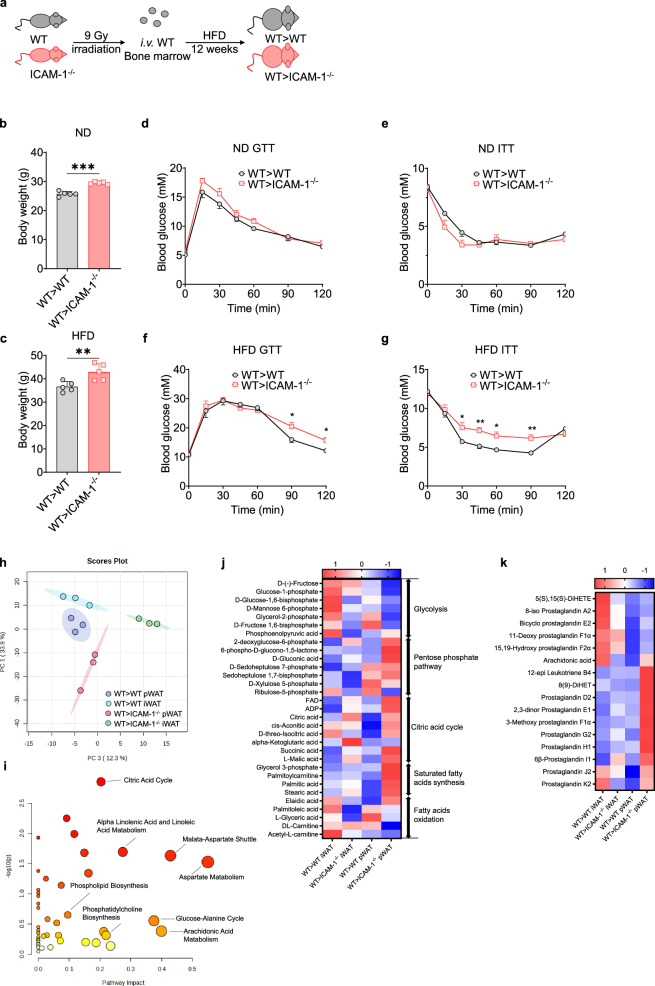
ICAM-1 deficiency in stromal cells leads to hyperplastic obesity and impaired insulin sensitivity. **a** Schematic diagram of the experimental protocol used to reconstitute ICAM-1^−/−^ mice and their wild-type (WT) littermates with WT bone marrow, followed by HFD treatment. **b-c** The body weights of WT > WT and WT > ICAM-1^−/−^ mice after being fed ND or HFD for 12 weeks. Error bars represent means ± SD. **p < 0.01, ***p < 0.001. Representative of two independent experiments. **d**–**g** Glucose-tolerance test (**d**, **f**) and insulin-tolerance test (**e**, **g**) of ND (**d**, **e**) or HFD-treated (**f**, **g**) WT > WT and WT > ICAM-1^−/−^ mice (*n* = 5 mice in each group). Error bars represent means ± SEM. *p < 0.05, **p < 0.01. Representative of two independent experiments. **h** Principal component analysis of the metabolites in perigonadal (pWAT) and inguinal white adipose tissue (iWAT) of HFD-treated WT > WT and WT > ICAM-1^−/−^ mice. **i** Metabolomic pathway analysis of the differential metabolites in perigonadal adipose tissue of HFD-treated WT > WT and WT > ICAM-1^−/−^ mice (*n* = 3 mice in each group). **j-k** Heatmaps displaying differential metabolites involved in metabolic processes of glycolysis, pentose phosphate pathway, citric acid cycle, saturated fatty acids synthesis, and fatty acids oxidation (**j**), along with those involved in eicosanoid metabolism (**k**) in inguinal (iWAT) and perigonadal white adipose tissue (pWAT) of HFD-treated WT > WT and WT > ICAM-1^−/−^ mice (*n* = 3).

We then questioned whether ICAM-1 deficiency in preadipocytes could regulate obesity and its related insulin resistance. Previous studies have demonstrated that ICAM-1 deficient mice were more susceptible to obesity [32, 33]. However, ICAM-1 is widely expressed in immune cells and stromal cells, including preadipocytes, making it difficult to determine which specific cell type with ICAM-1 deficiency contributes to obesity. To verify the role of ICAM-1 in stromal cells, we reconstituted lethally irradiated ICAM-1 deficient mice and their ICAM-1 sufficient littermates with wild-type bone marrow (Fig. 5a, S6a). We found that mice with ICAM-1 deficiency in stromal cells were prone to obesity, especially upon HFD-treatment (Fig. 5b, c). An increase in white adipose tissue but not lean body mass was observed (Fig. S6b, c), suggesting that ICAM-1 in stromal cells plays a crucial role in restraining obese adipose tissue remodeling. To evaluate the contribution of adipocyte hypertrophy and adipocyte hyperplasia to the obese adipose tissue, we measured the adipocyte size and found that enhanced obesity in mice with ICAM-1 deficiency in stromal cells was not related to hypertrophy of adipocytes (Fig. S6d, e). These results demonstrate that mice with ICAM-1 deficiency in stromal cells exhibit a strong ability in generating new adipocytes during obesity development.

We also assessed the metabolic status of mice lacking ICAM-1 specifically in stromal cells by conducting glucose tolerance tests (GTT) and insulin tolerance tests (ITT). Those mice showed worsened insulin resistance compared to WT mice when subjected to an HFD but not normal feeding conditions (Fig. 5d–g). These findings were consistent with earlier studies showing that ICAM-1 knockout mice fed on an HFD exhibited elevated plasma glucose and serum insulin levels [32, 33]. To gain a deeper understanding of the metabolic alterations in adipose tissue of mice with ICAM-1 deficiency in stromal cells, we conducted a metabolomics analysis on adipose tissue collected from these bone marrow reconstituted mice. In the PCA analysis of metabolites in perigonadal (pWAT) and inguinal white adipose tissues (iWAT) from WT > ICAM-1^−/−^ and WT > WT mice, there were differences between WT > ICAM-1^−/−^ and WT > WT adipose tissues, with the most significant difference observed in the pWATs (Fig. 5h, S6f). We detected 138 differential metabolites when comparing the metabolites in visceral adipose tissues with those in the other two groups. As expected, the pathways related to glucose and lipid metabolism, mitochondrial electron transport chain, metabolism of linoleic and arachidonic acid, catecholamine synthesis, and synthesis of phospholipids, particularly phosphatidylcholine, were significantly enriched (Fig. 5i, S6g). The abundance of metabolites of the classic glycolysis pathway in the pWAT of WT > ICAM-1^−/−^ mice was lower than that in WT > WT mice (Fig. 5j). Of note, the metabolites in the pentose phosphate pathway were found to be enriched in the pWAT of the WT > ICAM-1^−/−^ mice (Fig. 5j). This pathway generates NADPH, the major source of reducing power, which can be utilized in the synthesis of fatty acids and sterols in cells. Meanwhile, the accumulation of intermediate metabolites in the citric acid cycle, such as citric acid, can be observed in pWAT of the WT > ICAM-1^−/−^ mice (Fig. 5j). Citric acid can be shuttled across the mitochondrial membrane into the cytoplasm and broken down by the enzyme citrate lyase into oxaloacetate and acetyl-CoA. The latter is a precursor for fatty acid synthesis in the cytoplasm [34]. Consistently, an increase in the metabolites of the fatty acid synthesis pathway was observed (Fig. 5j). Importantly, these significant differences in the pWAT between WT > WT and WT > ICAM-1^−/−^ mice were not sharp in the iWAT. Thus, lacking ICAM-1 in stromal cells, visceral adipose tissues actively convert the energy into fatty acids for storage. The increased lipid accumulation in visceral adipose tissue could result in enhanced insulin resistance in mice with ICAM-1 deficiency in stromal cells.

We further compared the differences in the metabolic profiles of ICAM-1^−/−^ and WT adipose tissue against the reported metabolite “fingerprint” of in vitro adipogenesis. In addition to the accumulation of citric acid intermediates, the differentiated primary adipocytes show an increase in odd-chain fatty acid [35, 36]. We found an enrichment of odd-chain fatty acids, specifically pentadecanoic acid (C15) and undecanedioic acid (C11), in ICAM-1 deficient adipose tissues (Fig. S6f). Fatty acid desaturation and eicosanoid biosynthesis have been implicated as vital to the adipogenic differentiation process [35, 37, 38]. The concentrations of polyunsaturated fatty acids (PUFAs) and the eicosanoids were significantly enriched in pWAT of ICAM-1 stromal-deficient mice (Figs. S6h, 5k). Eicosanoid lipid mediators, such as prostaglandins and leukotrienes, are potent regulators of vascular dynamics, platelet aggregation, chemotaxis, and inflammation, and could be closely related to the metabolic disorder observed in ICAM-1 deficient mice. These differences between genotypes were more pronounced in pWATs compared to iWATs, likely due to higher adipogenesis rates in visceral adipose tissue [39]. Therefore, ICAM-1 deficiency in stromal cells boosts adipogenesis and energy storage in adipose tissue, suggesting that ICAM-1 restrains adipogenesis and maintains energy homeostasis.

### ICAM-1-mediated adhesion of immune cells to preadipocytes is associated with obesity in humans

**Fig. 6:**
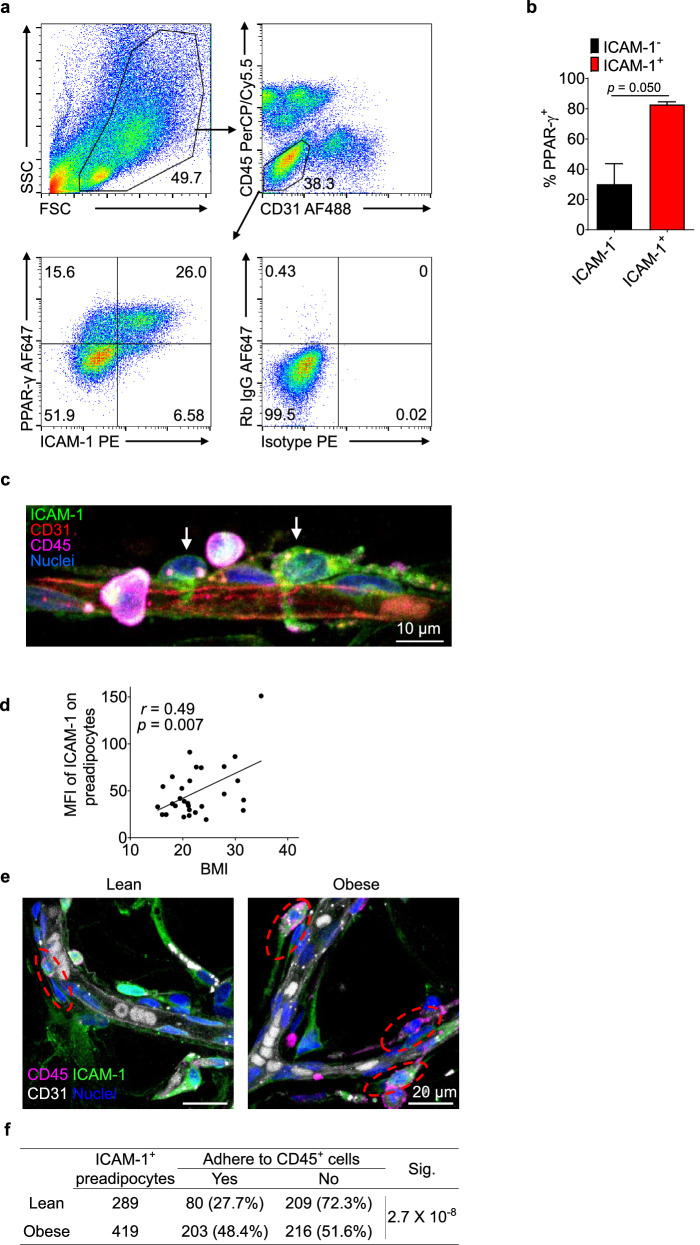
Human preadipocytes are ICAM-1^+^ and adhere to immune cells. **a**–**b** Representative plots (**a**) and statistical analysis (**b**) of flow cytometry showing PPAR-γ is predominantly expressed by CD31-CD45-ICAM-1^+^ preadipocytes in human adipose tissue (*n* = 3). Means ± SEM were shown in **b**. Paired, two-tailed *t*-test was performed. **c** Representative fluorescent micrograph showing the adherence of CD45^+^ immune cells to perivascular CD31^−^CD45^−^ICAM-1^+^ preadipocytes in human adipose tissue. **d** Expression of ICAM-1 on CD31^−^CD45^−^ICAM-1^+^ preadipocytes of human adipose tissues was analyzed by flow cytometry. Pearson’s correlation of ICAM-1 expression and BMI of subjects is shown (*n* = 29). **e**–**f** Representative fluorescent micrographs (**e**) and statistics (**f**) of adhesion between CD45^+^ immune cells and CD45^−^CD31^−^ICAM-1^+^ preadipocytes in adipose tissue of lean and obese subjects. Cell enumeration was performed on confocal images of whole-mount staining of adipose tissue obtained from three lean donors (with BMIs of 21.0, 21.0, and 21.1) and three obese donors (with BMIs of 27.9, 30.5, and 31.5) (about 20 representative image files per donor). *p* value was obtained by two-tailed Fisher’s exact test.

To assess the significance of our findings based on mouse preadipocytes in human preadipocytes, we examined ICAM-1 expression in human adipose tissue. We found that ICAM-1 is also expressed on a population of CD31^−^CD45^−^ SVF cells isolated from freshly collected adipose tissue of human subjects undergoing plastic surgery (Fig. 6a and Table S4). Interestingly, these ICAM-1^+^ ASCs similarly expressed PPAR-γ (Fig. 6a, b), demonstrating that they are preadipocytes with the potential to undergo adipogenesis. These results are consistent with that of a scRNA-seq study of stromal cell populations from human adipose tissue [9]. Similar to observations in the mouse adipose tissue, these human ICAM-1^+^ preadipocytes were also found in close proximity to blood vessels as well as in contact with CD45^+^ immune cells (Fig. 6c). We then examined ICAM-1^+^ preadipocytes from adipose tissue of several human subjects and found that the ICAM-1 expression on CD31^−^CD45^−^ SVF cells was positively correlated with the subjects’ body-mass index (BMI) (Figs. 6d, S7a), mirroring the increased ICAM-1 expression on preadipocytes in obese mice (Fig. 3d–f). Importantly, we observed a substantial increase in direct contacts between preadipocytes and CD45^+^ immune cells in obese subjects (Fig. 6e, f). This likely results from both heightened ICAM-1 expression on preadipocytes (Fig. 6d) and greater accumulation of CD45^+^ cells in adipose tissue of obese individuals (Fig. S7b). Our findings strongly imply that ICAM-1 could serve as a marker for human preadipocytes and might be involved in forming an immune niche for these cells in human adipose tissue.

## Discussion

Various investigations in in vitro experimental systems have delineated an exquisite regulatory network of adipogenesis. However, the identity, dynamics, and tissue niche of preadipocytes in vivo, particularly how they are modulated by immune components, are yet to be fully understood. In this study, we demonstrated that ICAM-1 is selectively expressed by committed preadipocytes. These ICAM-1^+^ preadipocytes reside perivascularly in both mouse and human adipose tissue. Under obese conditions, an abundance of immune cells adhere to preadipocytes via the ligation to ICAM-1 and restrain the ability of preadipocytes in generating adipocytes. In the absence of ICAM-1, PDGFR-a^+^ preadipocytes exhibit less adhesion with immune cells, resulting in enhanced adipogenesis. Taken together, ICAM-1 marks preadipocytes, sustains their immune microenvironment, and acts as a checkpoint for adipogenesis.

Over the past decade, significant advancements have been achieved in characterizing adipose progenitor cells in vivo, particularly through lineage tracing and single-cell RNA sequencing approaches. However, challenges persist in consistently identifying these stromal vascular fraction-derived cells across species and adipose depots, with ongoing debates about their differentiation plasticity and functional heterogeneity [5, 6, 40]. In our study, adipogenic competency analysis and in vivo tracing studies revealed that ICAM-1 is a reliable marker of preadipocytes. By using lineage tracing mice and immuno-staining of the adipose tissue, we revealed that these ICAM-1^+^ preadipocytes are located in close proximity to the vasculature. This location likely allows preadipocytes to easily sense the mitogenic or adipogenic cues carried in the blood. This aligns with an early seminal study showing that the PDGFR-β^+^ adipose progenitor cells, responsible for adipogenesis during the first postnatal month, reside within the mural cell compartment of the adipose vasculature. Notably, PDGFR-β was also selectively expressed by the ICAM-1^+^ ASCs. Furthermore, ICAM-1^+^ ASCs are present not only in adult mice but also in adipose tissue of P18 and P30 mice, where they express Fabp4 and differentiate into mature adipocytes. Together, these results suggest that the spatiotemporal expression of key preadipocyte markers, including ICAM-1 and PDGFR-β, remains consistent from early development into adulthood. The ICAM-1^+^ preadipocytes express a panel of chemokines and CAMs capable of attracting leukocytes and mediating their adhesion. Upon extensive analysis of adipose tissue from wild-type and lineage reporter mice, as well as adipose tissue from human subjects, we found that about 25% of the preadipocytes adhere to one or more immune cells in the naïve tissue; while this percentage can be as much as 50% in obese subjects. As one of the dominant CAMs, ICAM-1 plays an important role in this heterotypic adhesion in adipose tissue, especially during obesity development.

Immune cells have recently been recognized as key components of stem cell niches and potentially regulating stem cell behaviors. For example, the self-renewal and differentiation of hematopoietic stem cells, intestinal stem cells, and muscle stem cells are reported to be regulated by macrophages and subsets of T cells [41]. Although the accumulation of immune cells in adipose tissue has been extensively characterized, the connection between immune cells and preadipocytes has not been appreciated. Our study demonstrated direct interactions between immune cells and preadipocytes in vivo. This attachment presumably facilitates the fate regulation of preadipocytes by immune cells. A similar attachment-dependent regulation of progenitor fate was reported in hepatic progenitor cells. In damaged liver, these cells are always associated with a niche enriched in fibrillar collagen and laminin. Detachment from specific matrix proteins, like laminin, can trigger the differentiation of progenitor cells [42, 43]. Adipose tissue hosts a diverse array of immune cells, which provide a rich source of juxtacrine signals that can influence various aspects of preadipocyte dynamics. These immune cells produce numerous inflammatory cytokines, such as IFN-γ, TNF-α, and IL-1β. Aside from their roles in inflammation, these cytokines also function as anti-adipogenic factors [23, 24, 44]. In addition, factors secreted by adipose immune cells can promote the “beiging” of adipose tissue [45, 46]. However, the intricate microenvironment of obese adipose tissue, characterized by excessive ECM deposition, may impede the diffusion of these cytokines and limit their regulatory effect on adipogenesis [47–49]. In our study, we found direct physical interactions between immune cells and preadipocytes—mediated by ICAM-1 and its counterreceptors, which may also establish a microenvironment to facilitate the efficient cytokine-mediated regulation of preadipocyte fate. Therefore, our study supports the cellular and molecular foundation of this immune niche in regulating preadipocyte differentiation.

Adipogenesis has been considered as a two-phase process that involves the commitment of mesenchymal stem cells to the adipose lineage, forming preadipocytes, and subsequent terminal differentiation of these preadipocytes into mature adipocytes [2, 50]. Research involving in vivo carbon-14 birth-dating techniques (which track ^14^C integration into adipocyte genomic DNA) and stable isotope-labeled thymidine tracing in mouse fat cells has revealed that newly formed adipocytes originate from actively dividing progenitor cells [13, 39]. In preadipocyte cell lines and primary preadipocytes, growth arrest appears to be required for adipocyte differentiation, as cellular confluence is necessary for these types of adipogenesis [51]. However, upon the addition of adipogenic stimuli, it has been observed that growth-arrested 3T3-L1 preadipocytes can re-enter the cell cycle and undergo mitotic clonal expansion, followed by the expression of genes that drive the adipocyte phenotype [50–52]. Therefore, the proliferation capacity harbored in preadipocytes forms the basis for the clonal expansion of preadipocytes during adipogenesis. Using ICAM-1 reporter mice, we found that *Icam1*-expressing preadipocytes actively proliferate in adipose tissue, with their proliferation significantly accelerated during obesity. Moreover, *Fabp4*-expressing ICAM-1^+^ cells, corresponding to the newly generated adipocytes at a late stage of adipogenesis, have an even higher potential for entering mitosis. This in situ proliferation of preadipocytes and their progeny is consistent with the findings of in vitro clonal expansion during adipogenic induction of preadipocyte cell lines, suggesting that mitotic activity could occur in the immediate adipocyte precursors during adipogenesis. Yet, the key factors or signaling pathways in driving this process are largely open for future exploration.

We observed an impaired glucose homeostasis linked to the increased propensity to obesity in mice with ICAM-1 deficiency in stromal cells. This is in agreement with previous reports on ICAM-1 knockout mice: Dong et al. observed increased plasma glucose and insulin levels in ICAM-1^−/−^ mice fed an HFD [32]; Gregoire et al. detected an almost two-fold increase in serum insulin in ICAM-1^−/−^ mice as compared to wild-type mice at the early stage of HFD-treatment [33]. By analyzing the metabolomics of adipose tissue from WT mice and ICAM-1^−/−^ mice reconstituted with WT bone marrow, we found that lipid metabolism was more abnormal in mice lacking ICAM-1 in stromal cells, especially in visceral fat, which is closely linked to insulin resistance compared to subcutaneous adipose tissue. Unlike the partial ICAM-1 expression in stromal cells of subcutaneous WAT, most of the stromal cells in visceral WAT expressed ICAM-1. This difference in ICAM-1 expression between visceral and subcutaneous adipose tissue explains why metabolite changes are more pronounced in visceral WAT when ICAM-1 is absent. Thus, ICAM-1 plays a crucial role in regulating energy balance and adipogenesis.

Our findings demonstrate that ICAM-1 deficiency enhances in vivo adipogenesis of preadipocytes in mice. While ICAM-1 is widely expressed by immune cells and non-hematopoietic cells and plays a role in immunoregulation, we observed no significant difference in immune cell accumulation within adipose tissue between ICAM-1^−/−^ and WT obese mice. Bone marrow transplantation experiments revealed that ICAM-1 expression by non-hematopoietic cells is a critical regulator of adipogenesis. Furthermore, lineage tracing in ICAM-1^null^ mice provided additional evidence that ICAM-1 deficiency promotes preadipocyte adipogenesis. However, these results do not exclude the possibility that ICAM-1 expressed on non-hematopoietic cells other than preadipocytes may indirectly influence preadipocyte fate. To further elucidate the precise mechanisms, future studies employing preadipocyte-specific or perivascular-specific knockout of the *Icam1* gene, or targeted overexpression of ICAM-1 in preadipocytes, will be essential.

In summary, our results presented herein demonstrate that ICAM-1^+^ adipose stromal cells are committed preadipocytes, residing in a perivascular niche surrounded by immune cells. ICAM-1 plays a key role in the interaction between preadipocytes and nearby immune cells, and it negatively regulates preadipocyte differentiation. Targeting the interaction between preadipocytes and immune cells could offer potential strategies to fight obesity and related metabolic disorders.

## Materials and methods

### Ethics approval and consent to participate

All methods were performed in accordance with the relevant guidelines and regulations. All experimental protocols were approved by a named ethics committee. Specifically, the protocols for collecting and analyzing human adipose tissue were approved by the Ethics Committee of Soochow University (ECSU-201600020). Informed consent was obtained from all participants. All animal experiments were approved by the Institutional Animal Care and Use Committee of the Shanghai Institute of Nutrition and Health, Shanghai Institutes for Biological Sciences of Chinese Academy of Sciences (ER-SIBS-241415M).

### Animals

To generate mice with tamoxifen-inducible Cre recombinase under the control of *Icam1* promoter, we employed CRISPR knock-in technology utilizing homology-directed repair. A donor vector containing CreERT2-polyA, which disrupts *Icam1* transcription, was flanked by a 5’ homology arm (2.77 kb, homologous to upstream of the *Icam1* start codon) and a 3’ homology arm (2.34 kb, homologous to downstream of the exon 1 target site). This donor vector was co-injected with Cas9 mRNA and *Icam1*-targeting gRNA into C57BL/6 J zygotes. Founder (F0) mice were screened for knock-in alleles by long-range PCR and sequencing. Positive F0 mice were subsequently bred with C57BL/6 J mice to obtain heterozygous F1 offspring. *Fabp4*-Cre mice (B6.Cg-Tg(Fabp4-cre)1Rev/J, #005069), mTmG reporter mice (B6.129(Cg)-Gt(ROSA)26Sortm4(ACTB-tdTomato,-EGFP)Luo/J, #007676), ICAM-1^−/−^ mice (B6.129S4-*Icam1*^*tm1Jcgr*^/J, #002867), and GFP transgenic mice (C57BL/6-Tg(CAG-GFP)1Osb/J, #003291) were from Jackson Laboratory (Bar Harbor, ME). *Itgam*^−/−^ mice were provided by C.M. Ballantyne (Baylor College of Medicine, Houston, TX) [53] and backcrossed with C57BL/6 mice as previously described [31, 54]. Gender-matched littermates that were either wild-type or properly genetically modified were selected as controls.

Diet-induced obesity was accomplished by feeding with an HFD containing 60 kcal% fat (Research Diets, New Brunswick, NJ). Littermates of the target genotypes were randomly assigned to receive either HFD or ND. Experimental analysis of these mice was performed after feeding them with HFD for 10-16 weeks. Experimenters were not blinded to animal genotype or dietary treatment during the study.

Tamoxifen-induced activation of CreERT2 recombinase was initiated by administration of tamoxifen (Sigma-Aldrich, St. Louis, MO) dissolved in corn oil freshly prepared at 10 mg/ml. Mice received tamoxifen at 30 μg/g body weight or corn oil vehicle via intraperitoneal injection at specified time points.

For EdU incorporation, mice in our study received an intraperitoneal injection at a dosage of 10 µg/g mice. After 24 h, the mice were euthanized, and adipose tissue was collected for further analysis.

Glucose-tolerance tests were performed by intraperitoneal injection of 1 g glucose per kilogram of body weight, while insulin-tolerance tests were performed by intraperitoneal injection of 0.75 U insulin per kilogram of body weight on overnight-fasted mice. Blood glucose levels were monitored at designated time points.

### Human samples

Human subcutaneous adipose tissue samples (about 5 g) were collected at the time of plastic surgery and processed immediately. The information on age, gender, and BMI for these donors is presented in Table S4.

### Flow cytometric analysis and cell sorting

Mouse and human adipose tissues were isolated, cut into small pieces, rinsed with PBS, and digested with 2 mg/ml collagenase at 37 ^o^C until all cells separated. Mouse adipose tissue takes 1 h and human adipose tissue takes 2 h for digestion. The cell suspensions were centrifuged to get rid of adipocytes and filtered through 70-μm sieves to obtain stromal and vascular fraction (SVF) cells. Erythrocyte lysis was performed thereafter. The resulting SVF cells were incubated with anti-CD16/CD32 to block Fc receptors prior to surface molecule staining. The antibodies against cell surface markers include anti-mouse CD54 (ICAM-1) PE, anti-mouse CD45 FITC, anti-mouse CD45 PE, anti-mouse CD45 perCP-Cy5.5, anti-mouse CD45 APC, anti-mouse CD31 FITC, anti-mouse CD31 PE, anti-mouse CD31 APC, anti-mouse sca-1 PE, anti-mouse sca-1 perCP-Cy5.5, anti-mouse CD140a (PDGFR-α) PE, anti-mouse CD140a (PDGFR-α) APC, anti-mouse CD29 APC, anti-mouse F4/80 APC, anti-mouse Integrin α_M_ (CD11b) PerCP/Cy5.5, anti-GFP AF488 and anti-human CD45 PE were from ThermoFisher (San Diego, CA); anti-mouse CD54 Alexa fluor 647, anti-mouse CD45 pacific blue, anti-mouse Siglec-F PE, anti-mouse CD31 pacific blue, anti-mouse CD140a BV605, anti-mouse ICAM-1 PE/Cy7, anti-mouse Sca-1 BV785, anti-human CD31 Alexa fluor 488, and anti-Human CD54 (ICAM-1) Alexa fluor 647 were from Biolegend (San Diego, CA); and anti-mouse CD34 Alexa fluor 647 was from BD Biosciences (San Jose, CA). Intracellular human PPAR-γ staining was performed with fixation and permeabilization kits after surface antibody staining. PPARγ (C26H12) Rabbit mAb (Cell Signaling Technology, Danvers, MA) and Alexa fluor 647-conjugated donkey anti-rabbit IgG (Life Technologies, Carlsbad, CA) were used. Click-iT™ Plus EdU Alexa Fluor™ 647 Flow Cytometry Assay Kit was used for detection of EdU incorporation following the manufacture’s instruction.

### Immunofluorescence

Whole mount staining of adipose tissue was performed as described previously [22]. Briefly, adipose tissue was fixed with 4% paraformaldehyde for 24 h at 4 ^o^C. The samples were then permeabilized with 1% Triton X-100, and non-specific binding sites were blocked with 1% BSA and 3% FBS in PBS prior to antibody staining. The antibodies used including goat anti-mouse ICAM-1 (R&D Systems, Minneapolis, MN), rabbit anti-mouse CD31 (Abcam), mouse anti-human CD45 APC (eBioscience), mouse anti-human CD54 (ICAM-1) Alexa fluor 488 (Biolegend), Alexa Ffuor 633-conjugated donkey anti-goat IgG (Thermo Fisher Scientific), Alexa fluor 568-conjugated donkey anti-goat IgG (Thermo Fisher Scientific), Alexa fluor 488-conjugated donkey anti-rabbit IgG (Thermo Fisher Scientific), and Alexa fluor 568-conjugated donkey anti-rabbit IgG (Thermo Fisher Scientific). The staining pattern was reveled under confocal fluorescence microscopy (Cell Observer, ZEISS, Oberkochen, Germany) and representative micrographs were taken.

### RNA sequencing

The mature adipocytes, ICAM-1^+^GFP^+^, ICAM-1^+^GFP^−^, and ICAM-1^−^ subsets of CD31^−^CD45^−^Sca-1^+^ SVF cells were sorted from HFD-treated (at 16 weeks) *Fabp4*-Cre;mTmG mice (Fig. 2d), followed by RNA extraction. Total RNA from sorted cell subsets was treated with DNase I. Messenger RNA was enriched with Oligo (dT) magnetic beads and fragmented before reverse transcription and second-strand cDNA synthesis. The cDNA libraries were then purified, ligated with adaptors, and amplified. Quality of the cDNA libraries was evaluated (Agilient 2100 Bioanalyzer) prior to high-throughput sequencing (Illumina HiSeq 2000). Illumina Casava1.7 software was used for base calling. Sequenced reads were trimmed for adapter sequence and masked for low-complexity or low-quality sequence (Reads N rate < 10%, q20 > 90%). Trimmed sequence was mapped to mm8 whole genome using SOAP2.21. Reads per kilobase of exon per megabase of library size (RPKM) were further calculated. Raw reads were deposited on GEO database of NCBI (https://www.ncbi.nlm.nih.gov/geo/), with accession number GSE93697. Gene set enrichment analysis (GSEA) was performed with GSEA software (v4.3.2) [55] and using gene sets from Molecular Signatures Database (MSigDB) [56], including MH mouse-ortholog hallmark gene sets and M5 ontology gene sets.

### Spontaneous adipogenesis assay

For the competitive spontaneous differentiation, the ICAM-1^+^ and ICAM-1^−^ fraction of CD45^−^CD31^−^ SVF cells were sorted from both wild-type mice and GFP mice by FACS and mixed at 1:1 ratio. The cell mixture was grown in culture medium (DMEM, 1 mg/ml Glucose, Life Technologies) supplemented with 10% FBS (Life Technologies). The growth and formation of lipid droplets of these cells were monitored with fluorescence microscope at different time points.

### In vitro adhesion assay

The sorted CD31^−^CD45^−^ ASCs and CD31^+^CD45^−^ endothelial cells from HFD-treated ICAM-1^+/+^ and ICAM-1^−/−^ mice were seeded at 10^5^ / well in 96-well plate with culture medium (DMEM, 1 mg/ml Glucose, Life Technologies) supplemented with 10% FBS (Life Technologies). Splenocytes from wild-type mice were prepared, labeled with CFSE, and applied to the ASC and endothelial cell culture at 10^6^/well. After 20 min incubation at 37 ^o^C, the non-adhered splenocytes were washed off with 200 μl PBS. The wash step was repeated three times. The remaining CFSE signals, representing the cells-attached splenocytes, were collected using a fluorescence plate-reader with excitation wavelength of 488 nm and emission wavelength of 519 nm. Each well was measured at 16 different points and 8 wells were included in each group.

### Non-targeted metabolomics analysis

The inguinal and perigonadal adipose tissues collected from BM-reconstituted mice treated with HFD were extracted for metabolites and analyzed using liquid chromatography tandem mass spectrometry (LC-MS/MS). Molecular feature peaks were detected using high resolution mass spectrum (HRMS) detection technology and matched with mzCloud, mzVault, and MassList databases. The Compound Discoverer 3.1 software was used for raw data preprocessing. Metabolites with coefficient of variance (CV) less than 30% in quality control (QC) samples were retained as final identification results for further analysis. The MetaboAnalyst 5.0 platform was used for multivariate statistical analysis of differential metabolites.

### Quantitative real-time PCR

Total RNA was extracted from cells with either Trizol reagent (Thermo Fisher Scientific) or RNeasy kit (Qiagen, Hilden, Germany), and reverse transcription was performed with PrimeScript kit (TaKaRa, Kusatsu, Japan) following the manufacturers’ instructions. Quantitative real-time PCR was carried out with FastStart Universal SYBR Green (Roche Applied Science, Indianapolis, IN) on 7900HT Fast Real-time PCR system (Thermo Fisher Scientific). Oligonucleotide primers were as follows: *Actb*, 5‘-CCACGAGCGGTTCCGATG-3’, 5‘-GCCACAGGATTCCATACCCA-3’;*Cebpa*, 5‘-GAGCTGAGTGAGGCTCTCATTCT-3’, 5‘-TGGGAGGCAGACGAAAAAAC-3’; *Fabp4*, 5‘-ACACCGAGATTTCCTTCAAACTG-3’, 5‘-CCATCTAGGGTTATGATGCTCTTCA-3’; *Icam1*, 5‘-GTGATGCTCAGGTATCCATCCA-3’, 5‘-CACAGTTCTCAAAGCACAGCG-3’; *Pparg*, 5‘-CACAAGAGCTGACCCAATGGT-3’, 5‘-GATCGCACTTTGGTATTCTTGGA-3’; *Zfp423*, 5‘-TGGCCTGGGATTCCTCTGT-3’, 5‘-CTCTTGACTTGTCACGCTGTT-3’; *Vcam1*, 5‘-TGAACCCAAACAGAGGCAGAGT-3’, 5‘-GGTATCCCATCACTTGAGCAGG-3’; *Ccl2*, 5‘-TTAAAAACCTGGATCGGAACCAA-3’, 5‘-GCATTAGCTTCAGATTTACGGGT-3’; *Ccl7*, 5‘-GCTGCTTTCAGCATCCAAGTG-3’, 5‘-CCAGGGACACCGACTACTG-3’; *Cxcl1*, 5‘-CTGGGATTCACCTCAAGAACATC-3’, 5‘-CAGGGTCAAGGCAAGCCTC-3’; *Ccl11*, 5‘-GAATCACCAACAACAGATGCAC-3’, 5‘-ATCCTGGACCCACTTCTTCTT-3’.

### Analysis of single-cell RNA-sequencing dataset

To analyze the transcriptome of SVF cells at the single cell level, we retrieved a published dataset (GSE109774) [19], which analyzed the transcriptome of individual cells from multiple organs of mice, including adipose tissue. During our analysis, the differences of ASC transcriptomes from different genders were noted and anatomical sites were not negligible, so to be more precise in identifying the cell subsets, the data obtained in subcutaneous fat of male mice were chosen for further analysis. Whereas we did include female subjects in all our wet experiments.

The Seurat R package was employed for tSNE analysis and identification of marker genes. Cells with fewer than 1000 detected genes were excluded, and genes expressed by fewer than 3 cells in the expression matrix were also not included. The values were normalized, and the highly variable features were identified with the function ‘FindVariableFreatures’. The results were scaled and used as input for PCA (principal component analysis). The number of significant PCs was determined by the function ‘ElbowPlot’, and we chose PC 19 as a cutoff for clustering the cells and tSNE analysis. The ASC clusters were identified by excluding other lineages of cells with their specific marker genes, including *Cd3e*, *Cd19*, *Itgam*, and *Pecam1*. The marker genes of each of the three ASC clusters were identified with the function ‘FindMarkers’ by using the other two clusters as references. The tSNE plots, heatmaps, and violin plots were plotted with respective functions from this package.

All codes used for the above analysis will be provided upon reasonable request.

### Statistics of experimental results

Pilot experiments were performed to estimate the sample sizes required for achieving significant results with appropriate statistical tests. The repeats of individual experiments are denoted in each figure legend. Significance of pair-wise comparison was assessed using two-tailed unpaired Student’s *t*-test unless otherwise denoted in figure legends. For data groups with more than one independent variables, two-way ANOVA and Sidak’s multiple comparison test were applied. Statistical significance is set as *p* < 0.05. Data are shown as means ± SEM unless otherwise indicated.

## Supplementary information

Video S1

Video S2

Video S3

Video S4

Supplementary Figures

Supplementary Material Legends

Table S1

Table S2

Table S3

Table S4

Scan S1

## Data Availability

The data of bulk RNA sequencing of mature adipocytes (Adi), ICAM-1^+^GFP^+^(I^+^G^+^), ICAM-1^+^GFP^−^ (I^+^G^−^), and ICAM-1^−^ (I^−^) ASCs from inguinal adipose tissue of HFD-treated *Fabp4*-Cre;mTmT mice were deposited in Gene Expression Omnibus (GEO) database, with accession number GSE93697. The single-cell RNA sequencing data analyzed were from GSE109774 [12].
