# Supplementary material for: ICAM-1 identifies preadipocytes and restricts white adipogenesis by adhering immune cells
Source: Cell Death Differ. 2025 Aug 15;33(2):298–313. doi: 10.1038/s41418-025-01551-2 (PMC12881632; doi:10.1038/s41418-025-01551-2)
Supplement: Supplementary file 5 — Supplementary Figures [file 41418_2025_1551_MOESM5_ESM.pdf]

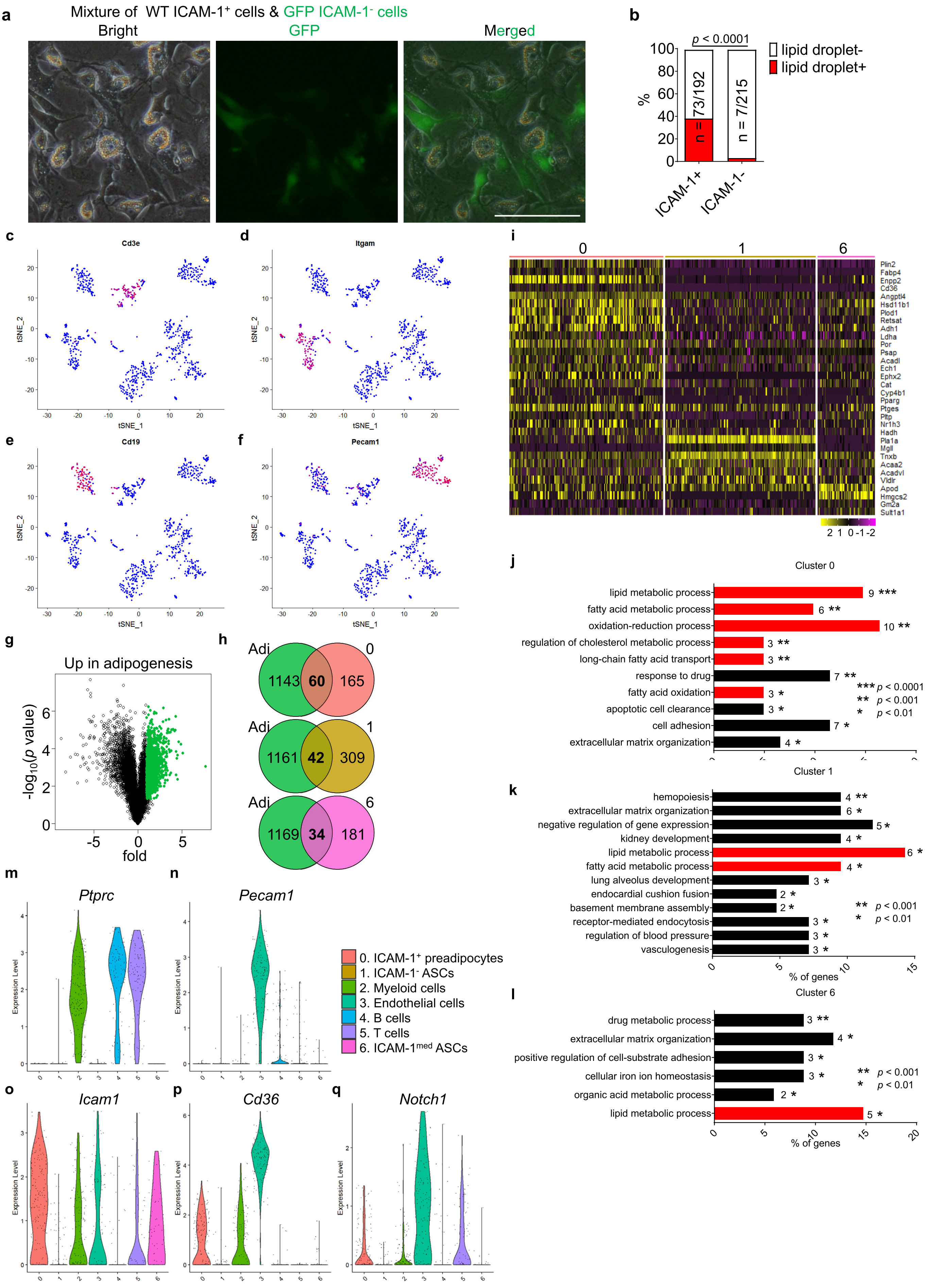

Figure S1. Competitive spontaneous differentiation and analysis of scRNA-seq of adipose SVF cells. Related to Fig. 1.

(legend continued on next page)

a-b. ICAM-1$^+$ ASCs spontaneously differentiate into adipocytes. ICAM-1$^+$ ASCs from wild-type mice and ICAM-1$^-$ ASCs from GFP-transgenic mice were co-cultured in regular medium and lipid droplets were observed and micrographs were taken on day 8 (**a**), Scale bars, 100 μM. The percentage of cells with or without lipid droplets in the co-culture was summarized from 2 independent experiments (**b**). P value was determined by two-tailed Fisher's exact test.

c-f. The expression of lineage markers in cells of tSNE clusters was analyzed, with *Cd3e* for T cells (**c**), *Itgam* for myeloid cells (**d**), *Cd19* for B cells (**e**), and *Pecam1* for endothelial cells (**f**).

g. Volcano plot showing the upregulated genes during adipogenesis of ASCs from a published microarray (GSE29899).

h. Venn plots showing the number of overlapping genes that both were highly expressed in the microarray of adipogenesis and in scRNA-seq of each ASC cluster.

i. Heatmap showing the adipogenic genes of each subset of ASCs, restrict to lipid metabolism-related pathways.

j-l. The functional categories of the overlapping genes, as indicated in **h**, between adipogenesis and cluster 0 (**j**), cluster 1 (**k**), and cluster 6 (**l**). Numbers and statistical significance of enriched genes were denoted on each bar. The categories of lipid and fatty acid metabolism-associated pathways were highlighted in red.

m-q. The expression levels of *Ptprc* (CD45) (**m**), *Pecam1* (CD31) (**n**), *Icam1* (**o**), *Cd36* (**p**), and *Notch1* (**q**) in each cluster of adipose SVF cells from the scRNA-seq dataset were shown in violin plots.

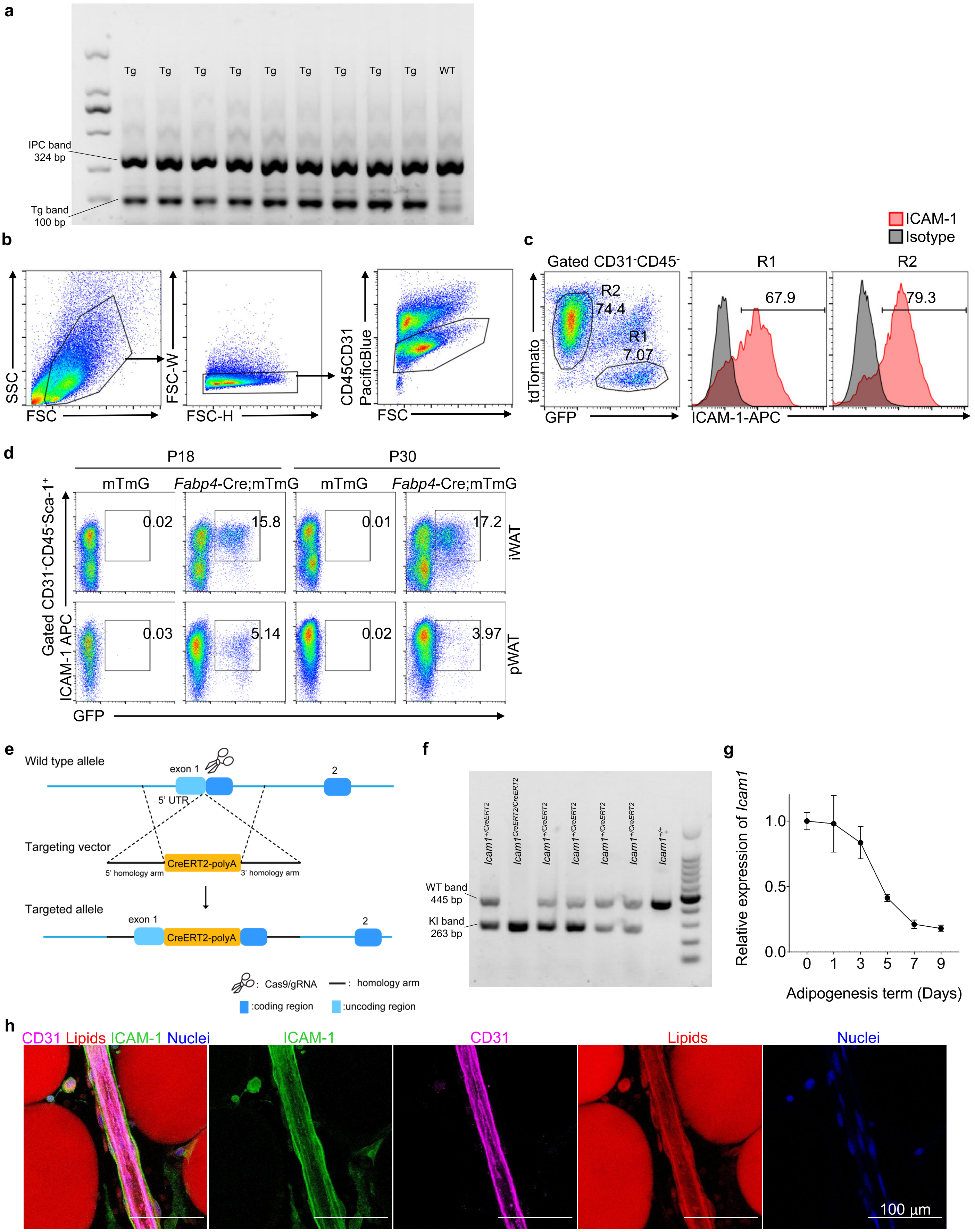

Figure S2. ICAM-1⁺ ASCs possess adipogenic potential. Related to Fig. 1.

a. Representative genotyping result of *Fabp4*-Cre mice is shown. Genotyping was performed using the standard PCR assay for generic Cre (JAX Protocol 22392). The expected band sizes were 100 bp for the transgene (Tg) and 324 bp for the internal positive control (IPC).

b. Gating strategies for CD31$^-$CD45$^-$ SVF cells from inguinal adipose tissue of *Fabp4*-Cre;mTmG mice.

(legend continued on next page)

c. Flow cytometric analysis of *Fabp4*-expressing $CD31^{-}CD45^{-}$ SVF cells in the perigonadal adipose tissue of *Fabp4*-Cre;mTmG mice. The majority of Fabp4-expressing cells are ICAM-1^{+}, representative of 5 independent experiments.

d. Flow cytometric analysis of ICAM-$1^{+}GFP^{+}$ preadipocytes in inguinal (iWAT) and perigonadal (pWAT) white adipose tissue isolated from P18 and P30 *Fabp4*-Cre;mTmG mice.

e. Strategy for generation of $Icam1^{+/CreERT2}$ mice. Cas9 mRNA, guide RNA, and a donor vector containing a 2.77 kb 5' homology arm, CreERT2-polyA, and a 2.34 kb 3' homology arm were microinjected into the zygote of C57BL/6J mice to generate F0 mice with CreERT2-polyA knock-in at the first exon of the *Icam1* locus.

f. Representative genotyping result from offspring of $Icam1^{+/CreERT2}$ mice is shown. The wild-type (WT) allele was amplified using a forward primer located 275 bp upstream of the *Icam1* start codon. The knock-in allele was detected using a forward primer positioned 123 bp upstream of the CreERT2-polyA cassette termination site. Both reactions employed a common reverse primer situated 103 bp downstream of *Icam1* exon 1.

g. Expression dynamics of *Icam1* during adipogenic induction of primary preadipocytes as detected by real-time PCR. Means ± SEM were shown (n = 4 wells for each time point).

h. Whole mount staining showing perivascular ICAM-1^{+} ASCs in HFD-treated mice. Adipocytes were counterstained with BODIPY, and it is important to note that they are negative for ICAM-1. Scale bars, 100 μM.

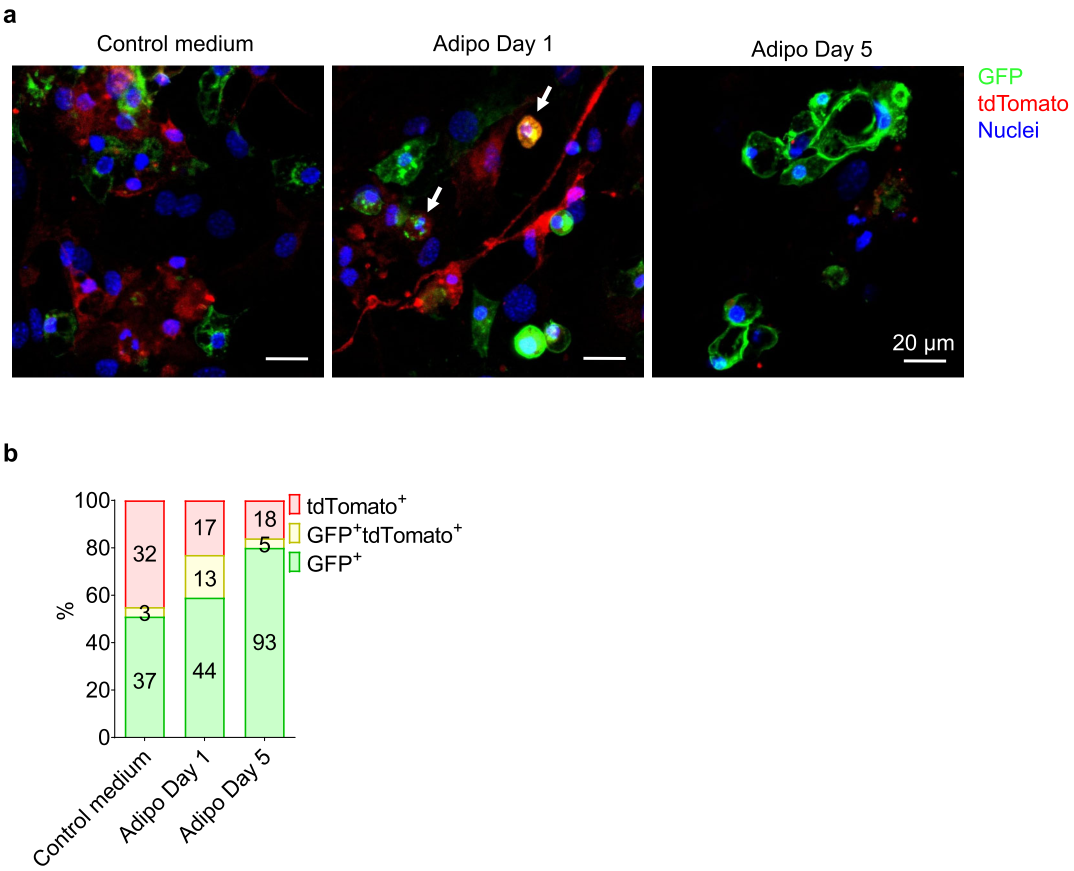

Figure S3. ICAM-1$^+$ preadipocytes transition to *Fabp4*-expressing incipient adipocytes during adipogenic induction. Related to Fig. 2.

a-b. Representative fluorescent micrograph (**a**) and quantification (**b**) of GFP$^+$, tdTomato$^+$, and double-positive ASCs. CD45$^-$CD31$^-$ICAM-1$^+$ SVF cells were sorted from the fat tissue of *Fabp4*-Cre;mTmG mice and treated with adipogenic induction medium. Arrows indicate the GFP$^+$tdTomato$^+$ incipient adipocytes. Three to four representative image files per condition were quantified. The numbers on the data bars denote the cell numbers of each population.

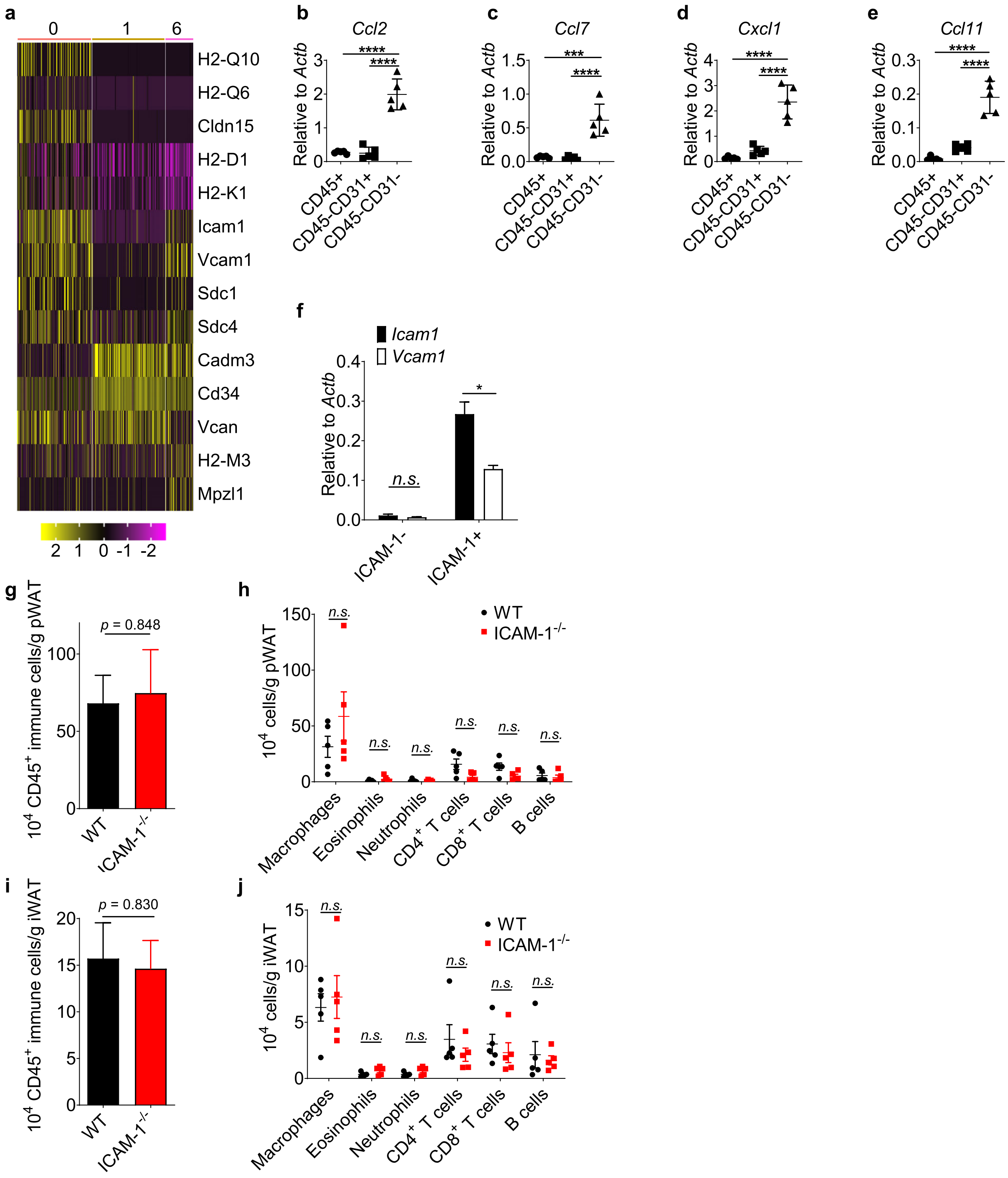

Figure S4. Committed preadipocytes have machinery for adhesion to immune cells. Related to Fig. 3.

a. The differentially expressed genes encoding cell adhesion molecules (CAMs) in clusters 0, 1, and 6 of ASCs, based on the scRNA-seq dataset.

b-e. The expression of chemokines for different immune cells, including *Ccl2* (**b**), *Ccl7* (**c**), *Cxcl1* (**d**), and *Ccl11* (**e**)**,** in $CD45^+$ immune cells, $CD45^-CD31^+$ endothelial cells, and $CD45^-CD31^-$ SVF cells from adipose tissue (n = 5 mice in each group). ***$p < 0.001$, ****$p < 0.0001$, ordinary one-way ANOVA followed by Sidak's multiple comparison test.

f. The mRNA levels of *Icam1* and *Vcam1* in sorted ICAM-1^- and ICAM-1^+ ASCs were analyzed by real-time PCR with *Actb* as reference (n = 3 in each group). Means ± SEM were shown. *$p < 0.05$, two-way ANOVA followed by Sidak's multiple comparison test.

g-j. The quantities of infiltrated total $CD45^+$ immune cells (**g, i**) and subsets of them (**h, i**), including macrophages, eosinophils, neutrophils, $CD4^+$ T cells, $CD8^+$ T cells, and B cells, in perigonadal (pWAT) (**g, h**) and inguinal white adipose tissue (iWAT) (**i, j**) of HFD-treated WT and ICAM-$1^{-/-}$ mice were analyzed by flow cytometry. Means ± SEM were shown. n = 5 mice in each group. Two-tailed, unpaired t test was performed for **g** and **i**; two-way ANOVA followed by Sidak's multiple comparison test was performed for **h** and **j**.

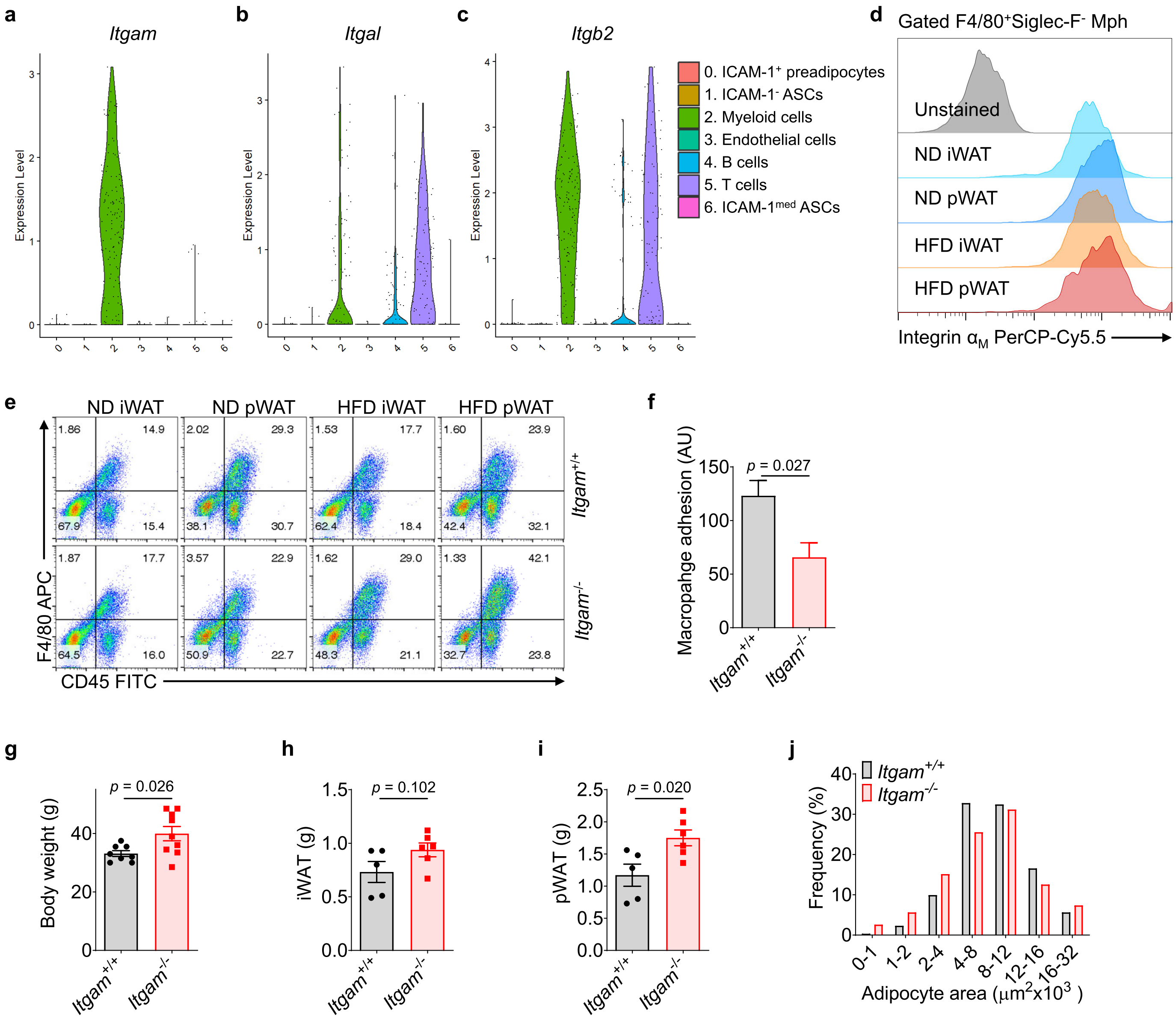

Figure S5. Integrin α_M deficiency reduces macrophage adhesion to preadipocytes and promotes adipocyte generation. Related to Fig. 4.

a-c. The expression levels of *Itgam* (**a**), *Itgal* (**b**), and *Itgb2* (**c**) in each cluster of adipose SVF cells from the scRNA-seq dataset were shown in violin plots.

d. The expression levels of integrin α_M on macrophages (F4/80$^+$Siglec-F$^-$) from inguinal (iWAT) and perigonadal white adipose tissue (pWAT) of ND or HFD-treated mice were analyzed by flow cytometry.

e. The percentages of CD45$^+$ immune cells and CD45$^+$F4/80$^+$ macrophages in inguinal (iWAT) and perigonadal white adipose tissue (pWAT) of ND or HFD-treated *Itgam*$^{+/+}$ mice and *Itgam*$^{-/-}$ mice were analyzed by flow cytometry.

f. *In vitro* macrophage adhesion assay showing that Integrin α_M is indispensable in the adhesion between macrophages and preadipocytes (n = 4 wells in each group). Means ± SEM were shown. Two-tailed, unpaired *t* test was applied.

g-j. The body weight (n = 8) (**g**), inguinal adipose tissue (n = 6) (**h**), perigonadal adipose tissue (n = 6) (**i**), and adipocyte size (**j**) of HFD-treated *Itgam*$^{+/+}$ mice and *Itgam*$^{-/-}$ mice. The adipocyte size was analyzed by measuring the area of 300 adipocytes from sections of adipose tissue of 4 mice in each group.

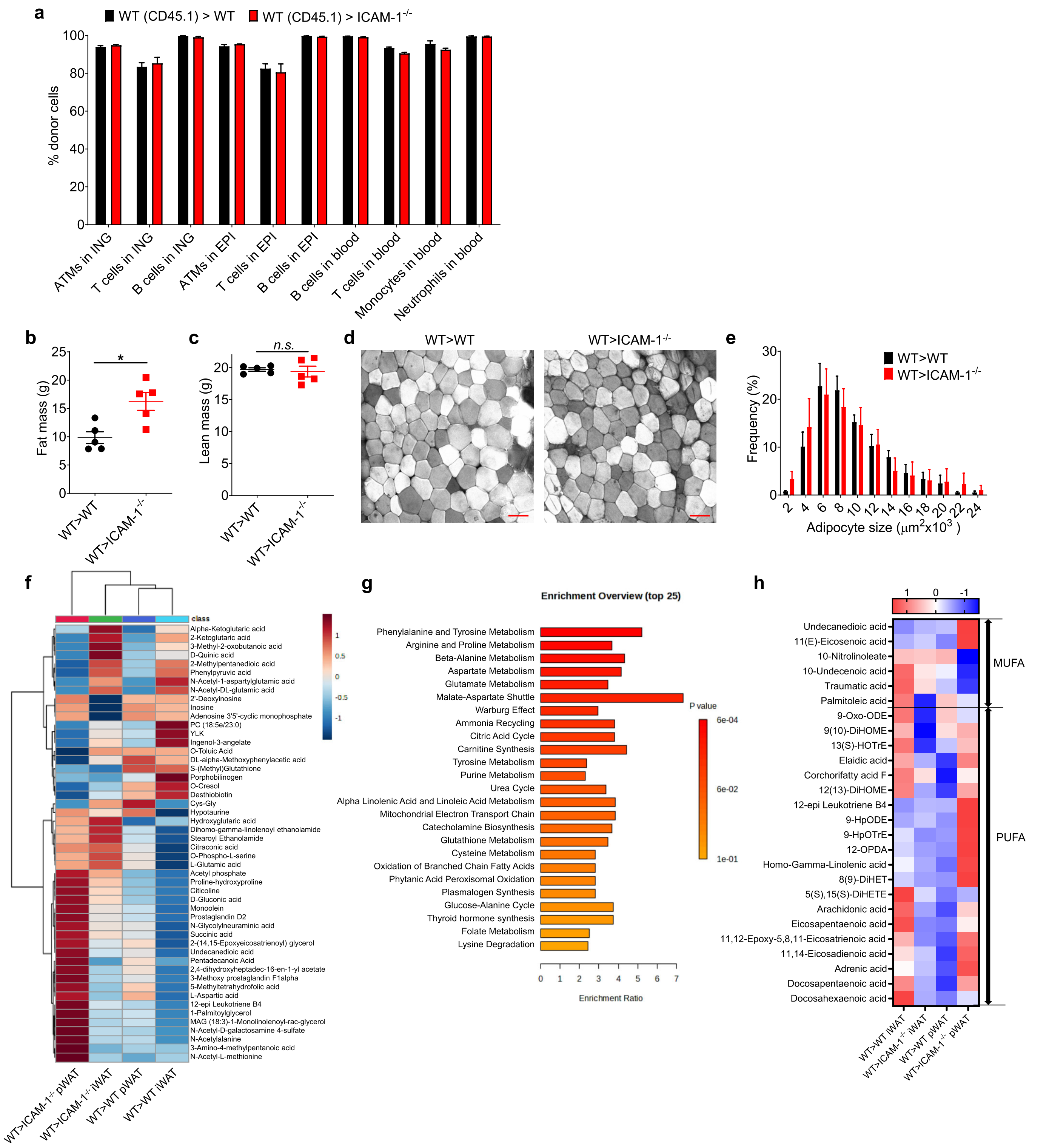

Figure S6. Deficiency of ICAM-1 in stromal cells promotes adipogenesis in obesity. Related to Fig. 5.

a. Percentage of donor cells in chimeric mice generated by bone marrow transplantation, including percentage of donor macrophages, T cells, B cells in inguinal and perigonadal adipose tissues, and percentage of donor T cells, B cells, monocytes, and neutrophils in peripheral blood of recipient mice.

b-c. Fat mass (**b**) and lean mass (**c**) of bone marrow-reconstituted and HFD-treated WT (WT>WT) and ICAM$^{-/-}$ (WT>ICAM-1$^{-/-}$) mice. Means ± SEM are shown. *p< 0.05.

d-e. Representative confocal optical sections (**d**) and distribution of adipocyte size (**e**) in inguinal adipose tissues from HFD-treated WT>WT and WT>ICAM-1$^{-/-}$ mice (n=4). Scale bars, 100 μM.

f. Heatmap showing the top 50 differential metabolites in perigonadal (pWAT) and inguinal white adipose tissue (iWAT) of HFD-treated WT>WT and WT>ICAM-1$^{-/-}$ mice (n = 3).

g. Overview of the top 25 metabolic pathways enriched in differential metabolites between perigonadal adipose tissues of HFD-treated WT>WT and WT>ICAM-1$^{-/-}$ mice (n = 3).

h. Heatmap displaying differential monounsaturated fatty acids (MUFA) and polyunsaturated fatty acids (PUFA) in perigonadal (pWAT) and inguinal white adipose tissue (iWAT) of HFD-treated WT>WT and WT>ICAM-1$^{-/-}$ mice (n = 3).

a

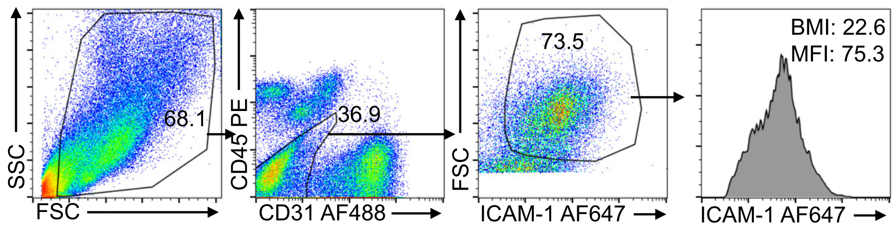

b

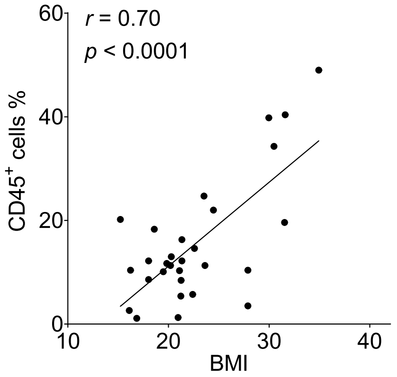

Figure S7. Immune cell infiltration in adipose tissue is correlated with BMI in human subjects. Related to Fig. 6.

a. Gating strategy for analysis of the expression level of ICAM-1 in $CD31^-CD45^-ICAM\text{-}1^+$ preadipocytes from adipose tissue of a representative human subject.

b. The percentages of $CD45^+$ immune cells in subcutaneous adipose tissue of human subjects show a significant correlation with their BMI (n = 29, Pearson's correlation coefficient was shown).
