# Supplementary material for: ICAM-1 identifies preadipocytes and restricts white adipogenesis by adhering immune cells
Source: Cell Death Differ. 2025 Aug 15;33(2):298–313. doi: 10.1038/s41418-025-01551-2 (PMC12881632; doi:10.1038/s41418-025-01551-2)
Supplement: Supplementary file 6 — Supplementary Material Legends [file 41418_2025_1551_MOESM6_ESM.pdf]

Supplementary Materials

Table S1. Marker genes of ASC clusters (0, 1 and 6). Related to Fig. 1.

Table S2. Cell surface marker genes of cluster 0. Related to Fig. 1.

Table S3. The interaction scores of significant ligand-receptor pairs between committed preadipocytes and stromal cells. Related to Fig. 4.

Table S4. Characteristics of adipose tissue donors. Related to Fig. 6.

Video S1, S2. The 3D projection of z-stack sections from inguinal (S1) and perigonadal (S2) adipose tissue in adult *Icam1-CreERT2;mTmG* mice treated with tamoxifen postnatally. Related to Figure 1.

Video S3, S4. The 3D projection of z-stack sections from inguinal (S3) and perigonadal (S4) adipose tissue in tamoxifen-treated, HFD-challenged *Icam1-CreERT2;mTmG* mice. Related to Figure 1.

Scan S1. Original western blot showing GAPDH (upper panel) and GFP under two exposure conditions (lower panel) in adipocytes isolated from *Icam1*$^{+/CreERT2}$;mTmG and *Icam1*$^{-/CreERT2}$;mTmG mice. Mice were pulsed with a tamoxifen-containing diet before induction of obesity with HFD. Related to Figure 4.
