# Supplementary figures and images for: ICAM-1 identifies preadipocytes and restricts white adipogenesis by adhering immune cells
Source: Cell Death Differ. 2025 Aug 15;33(2):298–313. doi: 10.1038/s41418-025-01551-2 (PMC12881632; doi:10.1038/s41418-025-01551-2)

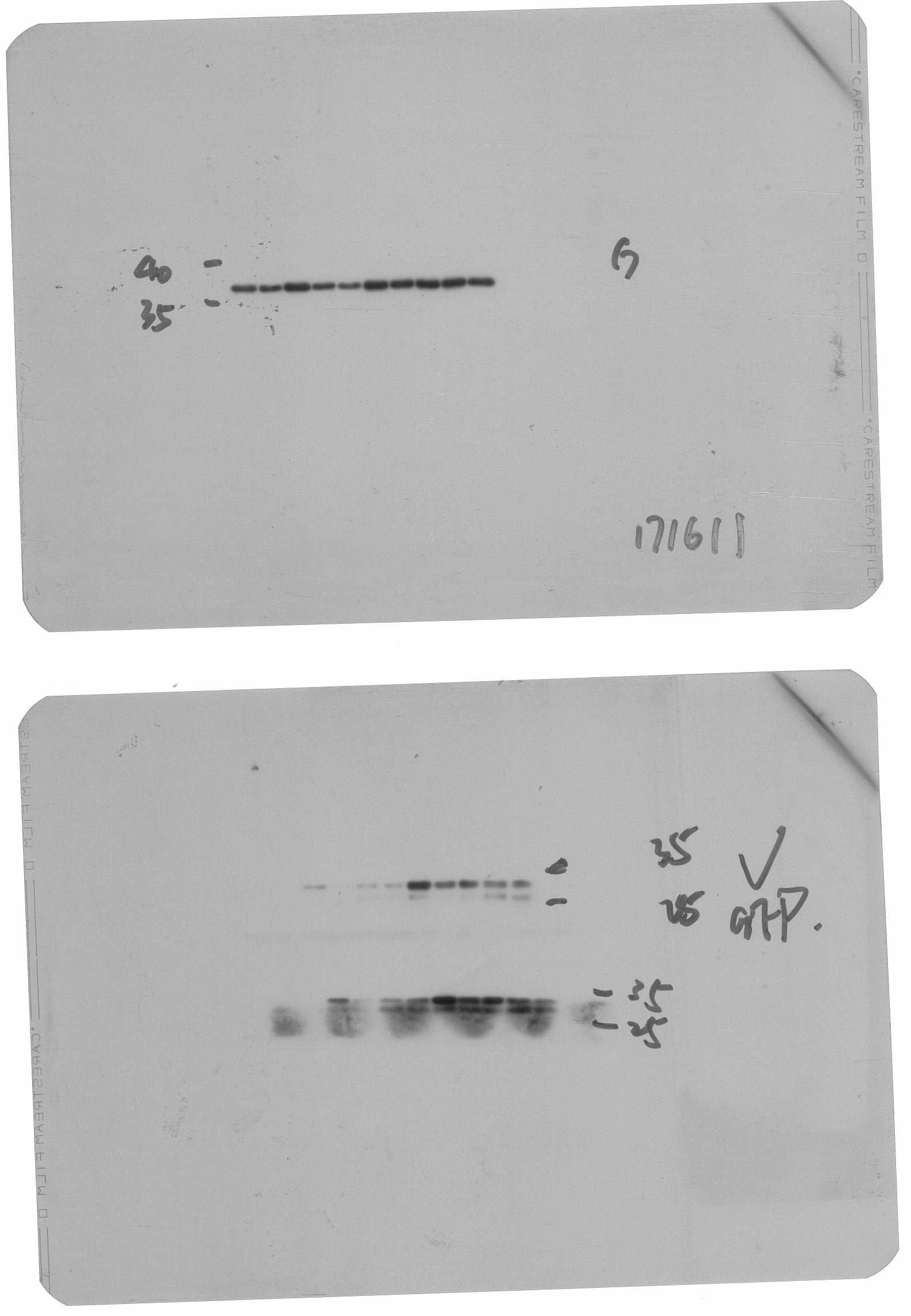

Supplement: Supplementary file 11 — Scan S1 [file 41418_2025_1551_MOESM11_ESM.tif]
